# The deubiquitinating enzyme complex BRISC regulates Aurora B activation via lysine-63-linked ubiquitination in mitosis

**DOI:** 10.1038/s42003-022-04299-4

**Published:** 2022-12-06

**Authors:** Qin Li, Yanfang Ma, Fen Chang, Yongjie Xu, Jingcheng Deng, Junyi Duan, Wei Jiang, Qihua He, Luzheng Xu, Lijun Zhong, Genze Shao, Li Li

**Affiliations:** 1 Department of Cell Biology, School of Basic Medical Sciences, Peking University Health Science Center, 100191 Beijing, China; 2 Department of Biochemistry and Biophysics, School of Basic Medical Sciences, Peking University Health Science Center, 100191 Beijing, China; 3 Center of Medical and Health Analysis, Peking University Health Science Center, 100191 Beijing, China

**Keywords:** Ubiquitylation, Mitosis

## Abstract

Faithful chromosome segregation requires bi-oriented kinetochore-microtubule attachment on the metaphase spindle. Aurora B kinase, the catalytic core of the chromosome passage complex (CPC), plays a crucial role in this process. Aurora B activation has widely been investigated in the context of protein phosphorylation. Here, we report that Aurora B is ubiquitinated in mitosis through lysine-63 ubiquitin chains (K63-Ub), which is required for its activation. Mutation of Aurora B at its primary K63 ubiquitin site inhibits its activation, reduces its kinase activity, and disrupts the association of Aurora B with other components of CPC, leading to severe mitotic defects and cell apoptosis. Moreover, we identify that BRCC36 isopeptidase complex (BRISC) is the K63-specific deubiquitinating enzyme for Aurora B. BRISC deficiency augments the accumulation of Aurora B K63-Ubs, leading to Aurora B hyperactivation and erroneous chromosome–microtubule attachments. These findings define the role of K63-linked ubiquitination in regulating Aurora B activation and provide a potential site for Aurora B-targeting drug design.

## Abstract (web summary)

The BRCC36 isopeptidase complex (BRISC) is identified as the deubiquitinating enzyme for Lys63-linked ubiquitination of Aurora B, and hyperactivated Aurora B in the absence of BRISC leads to erroneous chromosome–microtubule attachment during mitosis.

## Introduction

Mitosis is a complex and dynamic process precisely regulated by a variety of factors. Aurora B is an essential mitotic regulator, which belongs to the serine/threonine kinase family. Together with INCENP, Survivin, and Borealin, Aurora B forms the chromosomal passenger complex (CPC) to ensure accurate chromosome segregation^1^. Aurora B is targeted to different destinations during mitosis, where it regulates essential mitotic events. Aurora B localizes on chromosome arms in prophase and concentrates at inner centromeres from prometaphase to metaphase, where it corrects chromosome–microtubule attachment errors and activates the spindle assembly checkpoint (SAC). From anaphase onwards, Aurora-B transfers to the central spindle and accumulates at the midbody, regulating the contractile apparatus that drives cytokinesis^2,3^. Aurora B deficiency or inhibition results in severe chromosome segregation defects and polyploidy^4–7^. Overexpression of Aurora B has been detected in many human tumors^8–15^ and is closely associated with poor overall survival, adverse prognosis, and drug resistance^16,17^. Thus, Aurora B is a promising therapeutic target for cancer^18^.

Aurora B activation is tightly regulated at multiple levels, and the well-known mechanism is in *trans* phosphorylation. The binding of Aurora B with the IN box of INCENP activates low levels of kinase activity, which enables Aurora B to phosphorylate the TSS (Thr–Ser–Ser) motif in INCENP and triggers phosphorylation of Thr232 (T232) in its kinase domain, leading to its full activation^19–21^. Spatial and timely control in Aurora B activation is crucial for proper cell division^22^. In early mitosis, Aurora B kinase activity is high to phosphorylate multiple substrates at the kinetochore to destabilize erroneous kinetochore–microtubule attachments, and in later mitosis, activity decreases to ensure attachment stabilization^22,23^. How this regulation is achieved is worthy of intensive study^22^. In addition, Aurora B’s spatial- and time-specific dynamic localization is also essential for its function in mitotic progression. Recent evidence identified that monoubiquitylation of Aurora B by the E3 ubiquitin ligase cullin 3 (CUL3) regulates its removal from chromosome arms to the central spindle in anaphase^24,25^. However, it remains unknown whether there are other non-degradative ubiquitylation pathways for Aurora B and whether they could directly influence Aurora-B kinase activity. Identifying critical regulators of Aurora B activity is of prime importance.

Ubiquitination regulates protein stability as well as its function. Lysine63-linked ubiquitin chains (K63-Ubs) represent a particular ubiquitin topology that does not induce proteasome-dependent degradation but serves as a molecular platform for protein-protein interactions involved in receptor endocytosis, protein trafficking, and DNA damage repair^26–28^. Recent evidences show that conjugate to K63-linked ubiquitin chains is involved in the activation of kinases in AKT and ERK signaling pathways^27,29^.

BRCC36 (also named BRCC3, BRCA1/BRCA2-containing complex subunit 3) is a JAMM/MPN +-containing deubiquitinating enzyme (DUB), specifically cleaves K63-Ubs^30–33^. BRCC36 commonly exists in two complexes, the BRCA1-A complex and the BRCC36 isopeptidase complex (BRISC)^33,34^. The BRCA1-A complex consists of five proteins (Rap80, BRCC36, MERIT40/NBA1, BRE/BRCC45, and Abraxas1), specifically removing K63-Ubs on histones H2A and H2AX, antagonizing the RNF8-dependent ubiquitination at double-strand breaks^33,35–38^. BRISC complex localizes in the cytoplasm and consists of one unique component Abro1 (also called KIAA0157 or Abraxas2) and other three components (BRCC45/MERIT40/BRCC36) that are shared by the BRCA1-A complex^30,33,34^. Our previous work has demonstrated that BRISC is required for functional bipolar spindle assembly via deubiquitinating the spindle assembly factor NuMA at spindle pole^28^. However, whether BRISC-regulated K63-linked ubiquitination contributes to mitotic kinase activation has not been investigated so far. The similarity of the dynamic localization during mitosis between BRISC and Aurora B prompts us to identify its exact substrates involved in mitosis regulation.

Here, we report that Aurora B is modified by K63-Ubs in mitosis, which is correlated with its activation. We identify that the BRISC complex is the deubiquitinating enzyme for Aurora B, which could remove K63-Ubs from Aurora B to restrict its activation. We also provide evidence that Lys202 is the primary site for the K63-linked polyubiquitination of Aurora B, which is required for Aurora B activation and kinase activity, as well as for interaction with the CPC complex.

## Results

### Aurora B is modified by K63-linked polyubiquitination chains during mitosis

**Fig. 1:**
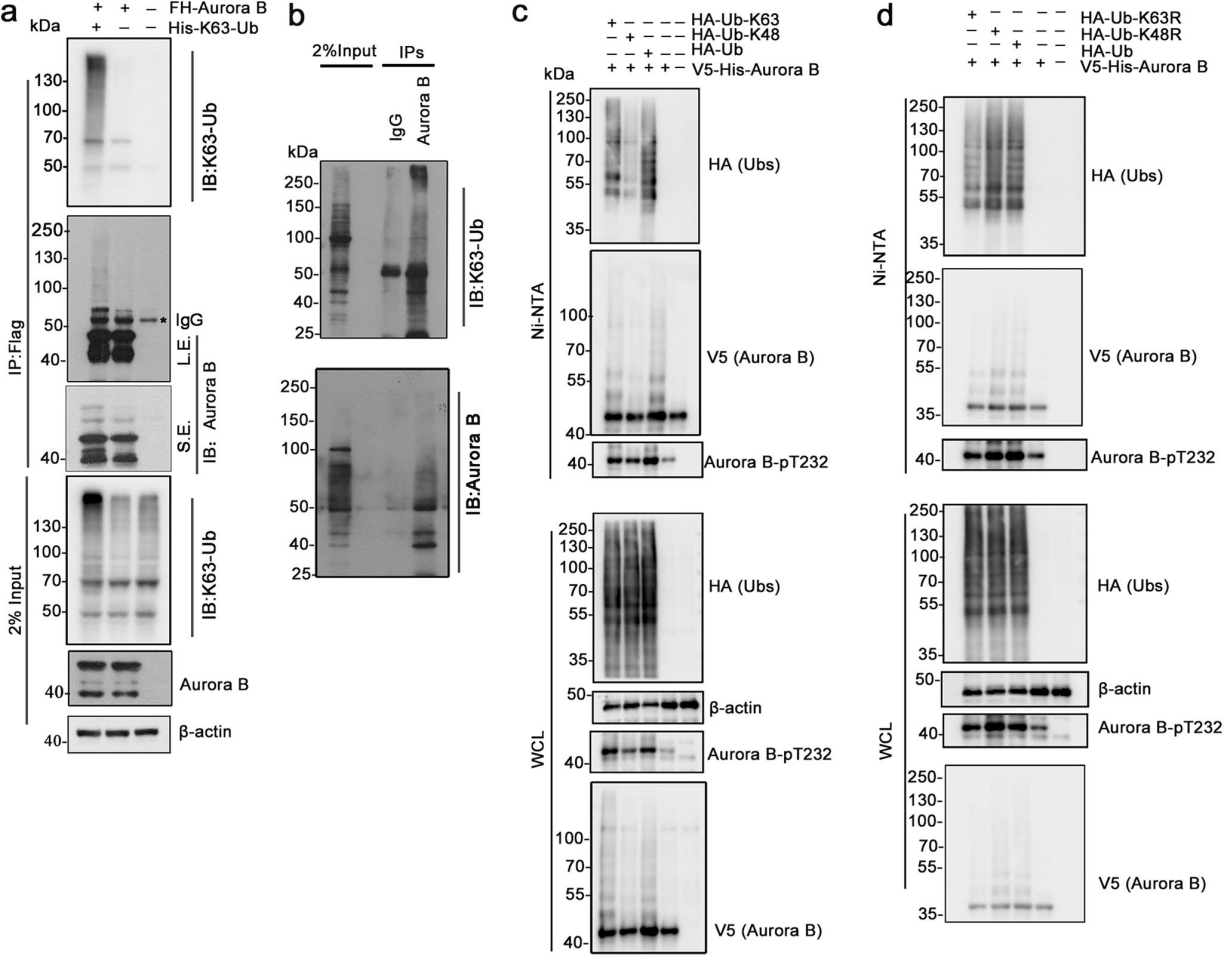
Aurora B is modified by K63-linked ubiquitination in mitosis. **a** K63-linked ubiquitination (K63-Ub) of ectopic Aurora B in mitotic HEK293T cells. Cells were co-transfected with Flag-HA-Aurora B (FH-Aurora B) and His-K63-Ub, and arrested in mitosis with 30 ng/mL nocodazole treatment for 16 h. Whole-cell lysates (WCL) were subjected to immunoprecipitation (IP) using anti–FLAG M2 agarose beads and IP samples were analyzed by immunoblotting (IB) using an antibody specifically recognizes K63-Ub chains. S.E., short exposure; L.E., long exposure. **b** K63-Ub modification of endogenous Aurora B in mitotic HeLa cells. HeLa cells were synchronized to mitotic (M) phase as described in (**a**), then subjected to IP using anti-Aurora B antibody followed by IB. **c**, **d** Confirmation of the K63-Ub modification of Aurora B under denaturing condition. HEK293T cells were transfected with V5-His-Aurora B and wild-type ubiquitin or with ubiquitin mutants containing only one Lys (**c**), Ub-K48R or Ub-K63R (**d**). Mitotic cells were subjected to Ni-NTA pull-down under denaturing conditions and the immunoprecipitates were analyzed by indicated antibodies.

To investigate the potential role of K63-linked ubiquitination in regulating the function of Aurora B, we first examined its ubiquitination in HEK293T cells. Since Aurora B expression peaks at the G2/M transition and its kinase activity is maximal during mitosis^39^, we synchronized cells to the mitotic phase by nocodazole (NOC) treatment^28^. HEK293T cells co-transfected with Flag-HA-Aurora B (FH-Aurora B) and His-K63-Ub, a ubiquitin mutant that only mediates K63-conjugated ubiquitin chains, were used for Flag-immunoprecipitation. The K63-linked polyubiquitination of Aurora B was detected with an antibody that specifically recognizes K63-Ub. As shown in Fig. 1a, K63-Ub species showed as several bands below 70 kDa, and a smear above 70 kDa. In addition, they are stronger in cells co-transfected with FH-Aurora B and His-K63-Ub than with FH-Aurora B alone. Moreover, a similar Aurora B pattern was obtained when the blot was probed with an anti-Aurora B antibody. Consistently, K63-Ubs-modified species could also be detected in the immunoprecipitates of endogenous Aurora B from mitotic HeLa cells (Fig. 1b), indicating that Aurora B could be ubiquitinated through a K63-linkage at M phase. To further verify this, we performed immunoprecipitation under denaturing conditions using ni-nitrilotriacetic acid (Ni-NTA) pull-down assay in HEK293T cells co-transfected with V5-His-Aurora B and wild-type ubiquitin or different ubiquitin mutants. As shown, K63-conjugated polyubiquitin chains of Aurora B were increased in cells transfected with the HA-Ub-K63-only, but not the HA-Ub-K48-only construct, in which only one Lys residue was retained while the rest of them were replaced with Arg residues (Fig. 1c). Consistently, K63-Ub chains of Aurora B was decreased in cells transfected with HA-Ub-K63R, an ubiquitin mutant in which K63 were replaced with Arg, but not with HA-Ub-K48R (Fig. 1d). These results indicate that Aurora B could be modified by K63-linked ubiquitination during mitosis.

### Aurora B associates with the deubiquitinating enzyme complex BRISC

To identify factors that regulate the K63-linked ubiquitination of Aurora B, we performed liquid chromatography-tandem mass spectrometry analysis using Flag-Aurora B-specific complex purified from HEK293T cells ectopically expressing Flag-Aurora B with or without K63-Ub. INCENP and Borealin, two CPC components, were detected in the Flag-Aurora B precipitates (Supplementary Fig. 1a and the Supplementary Data 1). Interestingly, BRCC36 and Abro1 were captured concomitantly when His-K63-Ub was co-transfected in HEK293T cells (Supplementary Fig. 1a and the Supplementary Data 1). BRCC36 and Abro1 are the two essential subunits of the BRISC complex. BRCC36 is a JAMM/MPN + domain containing K63-Ub-specific DUB, its enzymatic activity requires interactions with the MPN -domain cotaining Abor1 of the BRISC complex. Moreover, Abro1 regulates the formation and cytoplasmic localization of the BRISC complex. This result suggests that BRISC might regulate the ubiquitination of Aurora B.

**Fig. 2:**
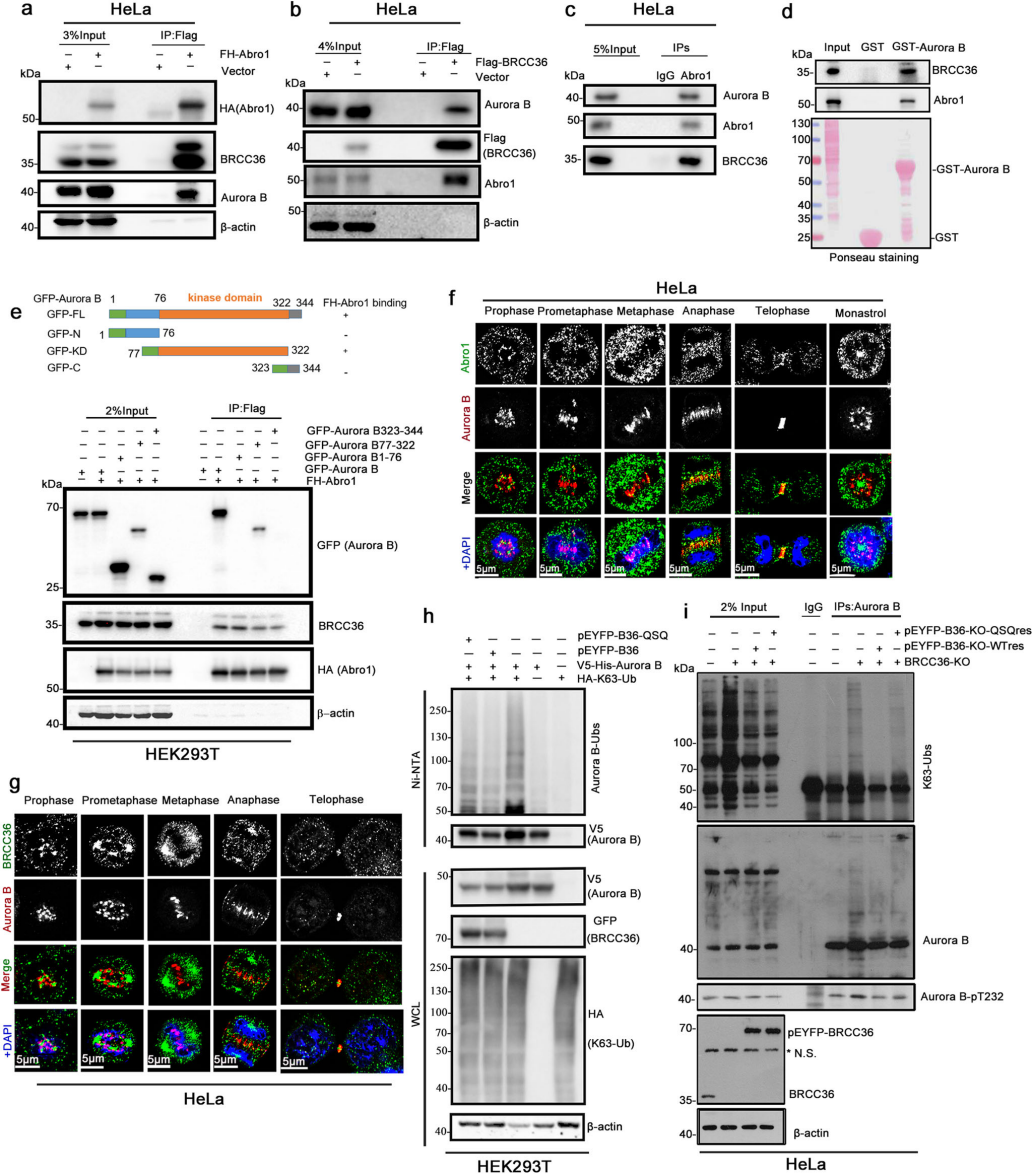
BRISC is the deubiquitinating enzyme for Aurora B in mitosis. **a**, **b** Flag-HA-Abro1 (FH-Abro1) or Flag-BRCC36 complex interacts with endogenous Aurora B in HeLa cells. Mitotic HeLa cells transfected with FH-Abro1 (**a**), Flag-BRCC36 (**b**) or vector control (Vector) were used for Flag-IP, followed by IB. **c** Endogenous interaction between Aurora B and Abro1/BRCC36 complex in HeLa cells. Immunoprecipitation was performed on mitotic HeLa cell lysates using an anti-Abro1 antibody. **d** Interaction between Aurora B and Abro1/BRCC36 complex in vitro. GST pull-down experiments were performed using GST or GST-Aurora B protein purified from bacteria and HeLa mitotic cell lysates. The GST-Aurora B binding protein was resolved by SDS–PAGE, visualized by Ponceau staining (bottom), and immunoblotting with the indicated antibodies (upper). **e** Identification of the Aurora B domain that binds Abro1. Upper, schematic diagram that represents the GFP-Aurora B truncated constructs. Lower panel, GFP-tagged Aurora B truncations were constructed. Flag-IP was performed in mitotic HEK293T cells transfected with the indicated plasmids. **f**, **g** Immunofluorescence staining was done in HeLa cells with the indicated antibodies to show localization of endogenous Aurora B (red), Abro1 (green) (**f**) or BRCC36 (green) (**g**). DNA was stained with DAPI. To observe the kinetochore localization of Aurora B, monopolar cells were generated with Eg5 inhibitor monastrol treatment. Bar, 5 μm. **h**. BRCC36 overexpression reduces Aurora B ubiquitination. HEK293T cells were transfected with the indicated plasmids, and the mitotic cell lysates were subjected to Ni-NTA pull-down under denaturing conditions, followed by immunoblotting with the indicated antibodies. **i** BRCC36 depletion-induced K63-Ub accumulation of Aurora B is repressed by restoration of BRCC36 WT but not BRCC36 QSQ, a DUB-inactive mutant of BRCC36. Endogenous Co-IP was performed in mitotic HeLa control cells or in BRCC36-knockout HeLa cells infected with or without the BRCC36 rescue virus, using an anti-Aurora B antibody.

To confirm the interaction between Aurora B and BRISC, we performed co-immunoprecipitation (Co-IP) assays. As shown, ectopic EGFP-Abor1 or EYFP-BRCC36 was detected in the Flag-Aurora B immunoprecipitates (Supplementary Fig. 1b, c). Endogenous Aurora B and BRCC36 were detected in the Flag-Abro1 immunoprecipitates (Fig. 2a), and endogenous Abro1 and Aurora B were detected in the Flag-BRCC36 immunoprecipitates (Fig. 2b). Furthermore, endogenous Aurora B and BRCC36 could also be pulled down by endogenous Abro1 in mitotic cells (Fig. 2c). GST pull-down assay revealed that GST-Aurora B interacts with BRCC36 and Abor1 in vitro (Fig. 2d). These results indicate that Aurora B binds to the BRCC36/Abor1-containing BRISC complex.

Next, to map the specific Abro1-binding region in Aurora B, we performed Co-IP experiments using lysates from HEK293T cells expressing full-length Flag-Abro1 and a pEGFP-tagged Aurora B fragment including the N-terminal domain (1–76), or the kinase-domain (77–322), or the C-terminal domain (323–344). The result showed that Aurora B binds to Abro1 with the kinase domain (Fig. 2e). In addition, we examined the subcellular localization of Aurora B and BRISC complex in mitotic HeLa cells. In agreement with the biochemical data, Aurora B partially co-localizes with Abro1 throughout mitosis, especially at the centromere, central spindle, and midbody (Fig. 2f). A similar co-localization pattern was also observed for Aurora B and BRCC36 (Fig. 2g). Together, these data indicate that Aurora B interacts with the BRISC complex during mitosis.

### BRISC regulates the K63-linked ubiquitination of Aurora B in mitosis

Since BRCC36 is a DUB, we attempted to investigate whether it could deubiquitinate Aurora B. An in vivo ubiquitination assay was performed using Ni-NTA pull-down under denaturing conditions. HEK293T cells were co-transfected with V5-His-Aurora B, HA-K63-Ub, and pEYFP-BRCC36, or pEYFP-BRCC36QSQ, a DUB-inactive mutant of BRCC36, in which two Zn^2+^-binding histidine residues (H122 and H124) were replaced by glutamine residues (Q122 and Q124)^28^. V5-His-Aurora B was then immunoprecipitated under denaturing conditions and analyzed by immunoblotting. The K63-linked ubiquitination of Aurora B was noticeably decreased by overexpression of BRCC36, but not the DUB-inactive BRCC36-QSQ (Fig. 2h). Consistent with this, endogenous K63-linked ubiquitination of Aurora B was increased in mitotic BRCC36 knockout (BRCC36-KO) HeLa cells. Moreover, BRCC36 deficiency-induced accumulation of K63-Ubs of Aurora B was reduced by the restoration of BRCC36 WT-res, but not BRCC36 QSQ-res (two sgRNA-resistant mutants) (Fig. 2i). Collectively, these data indicate that BRISC modulates the K63-conjugated ubiquitin chains of Aurora B, and Aurora B could be a substrate of BRISC in mitosis.

### BRISC deficiency leads to Aurora B hyperactivation and erroneous accumulation of chromosome–microtubule attachments

To gain an insight into the functional significance of BRISC-regulating ubiquitination of Aurora B, we first examined the protein stability of Aurora B in BRCC36 or Abro1 knockout HeLa cells, described as BRCC36-KO and Abro1-KO, respectively. The single guide RNA (sgRNA) targeting BRCC36 or Abro1 was designed according to the protocol from the website (https://chopchop.cbu.uib.no/) (Supplementary Fig. 2a), and the efficiency of the knockout was confirmed by Western blotting. Consistent with the previous report^40^, knockout of either BRCC36 or Abro1 dramatically disturbed the stability of the other one in the BRISC complex (Supplementary Fig. 2b), while the protein level of Aurora B was not obviously affected, as detected by Western blotting (Supplementary Fig. 2b) or immunofluorescence (IF) staining (Supplementary Fig. 2c, d).

**Fig. 3:**
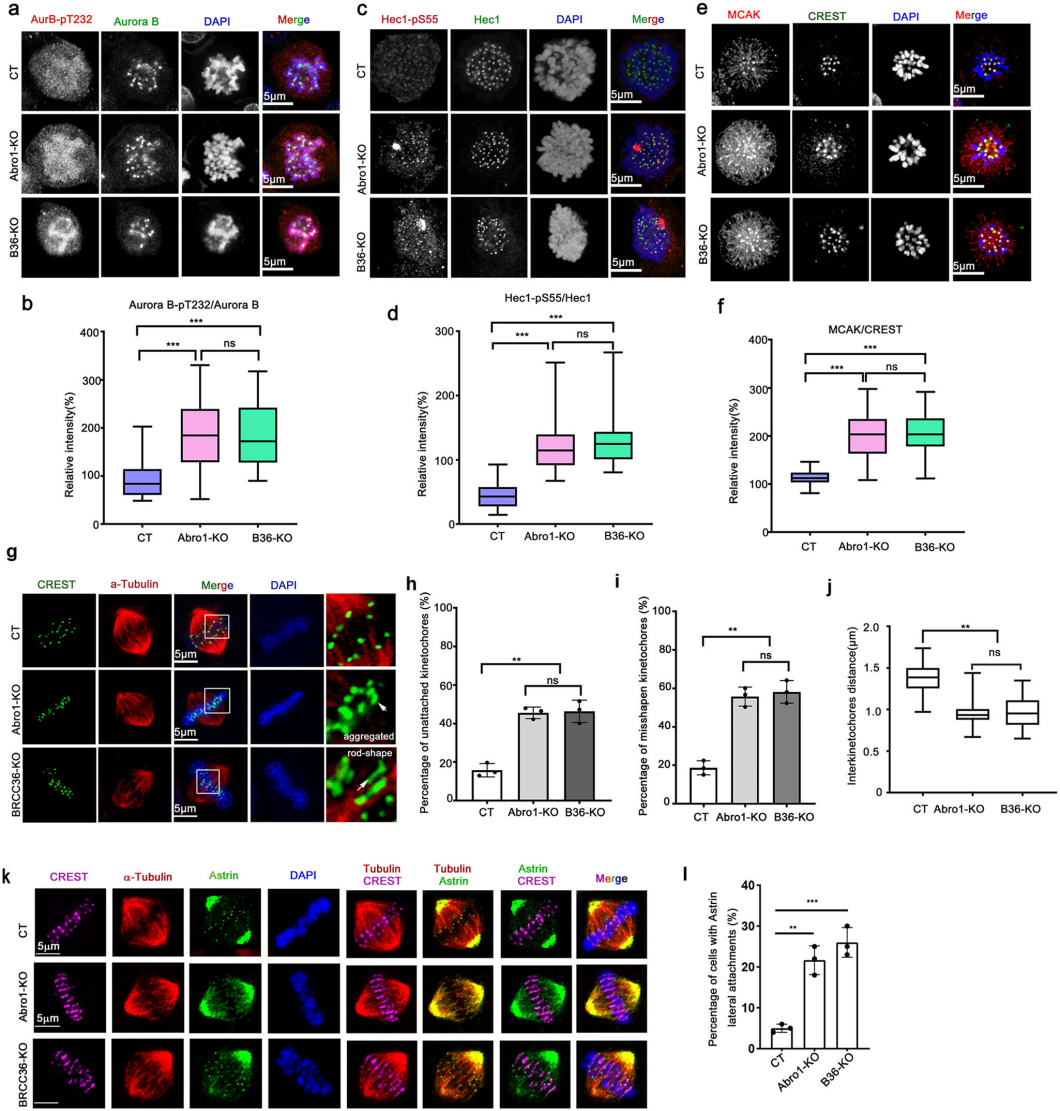
BRISC deficiency leads to Aurora B hyperactivation and erroneous kinetochore–microtubule (KT-MT) attachments. **a** Representative confocal images showing Aurora B activation in Abro1-knockout (Abro1-KO) or BRCC36-knockout (BRCC36-KO) HeLa cells. Cells were treated with 30 ng/mL nocodazole for 4 h and released into fresh medium for 30 min, fixed and stained with anti-Aurora B-pT232 (green) and anti-Aurora B (red) antibodies. DNA was stained with DAPI (blue). Bar, 5 μm. **b** Box-and-whiskers plots showing the quantification of the relative intensity of Aurora B-pT232 as compared with Aurora B, shown in **a**. Boxes show the upper and lower quartiles with a line at the median. Whiskers as the extent of 100% of the data. ***p < 0.001 versus CT, one-way ANOVA test, calculated with ZEN3.1 and GraphPad Prism 8 (*n* = 15 cells and 6 areas for each cell were counted). **c**, **e** Representative confocal images showing Aurora B catalytic activity on its substrates, Hec1 and MCAK, in Abro1-KO or BRCC36-KO HeLa cells. Cells were treated as described in **a** and stained with the indicated antibodies. Hec1 (green)；Hec1-pS55 (red)；MCAK (red); CREST (green); DNA (DAPI, blue). Bar, 5 μm. **d** Quantification of the relative intensity about Hec1-pS55, as compared with Hec1, shown in **c**. **f** Quantification of the relative intensity about MCAK, as normalized to the general kinetochore marker CREST, shown in **e**. **d**, **f** Boxes show the upper and lower quartiles with a line at the median. Whiskers as the extent of 100% of the data. ***p < 0.001 versus CT, one-way ANOVA test, calculated with ZEN3.1 and GraphPad Prism 8 (*n* = 15 cells and 6 areas for each cell were analyzed). **g** Representative confocal images showing abnormal KT-MT attachments in Abro1-KO or BRCC36-KO cells. HeLa cells were treated with double thymidine block, then released for 6 h, followed by 10 μM MG132 treatment for 4 h to prevent cells from metaphase to anaphase transition. The cells were fixed on ice to destabilize non-kinetochore microtubules and stained for CREST (green) and α-tubulin (red). Bar, 5 μm. **h** The percentage of paired kinetochores not attached to microtubules in all counted KT-MT connections. **i** The percentage of m**i**sshapen (aggregated or rod-shaped) kinetochores in all counted paired kinetochores. **h**, **i** Data are shown as means ± SD, *n* = 3 independent experiments. In each independent experiment, 30 cells, five paired kinetochores per cell were counted. **p < 0.01 versus CT, Student’s *t*-test. **j** Quantification of the interkinetochore distances shown in Supplementary Fig. 3a. *n* = 20 cells and five paired kinetochores for each cell were counted. **p < 0.01 versus CT, One-way ordinary ANOVA test. **k** Representative confocal images showing lateral KT-MT attachment in Abro1-KO or BRCC36-KO cells. HeLa cells were treated as described in **g**. α-tubulin, red; Astrin, green；CREST, purple; and DNA (DAPI, blue). Bar, 5 μm. **l** The percentage of cells showing Astrin lateral attachments. Data are shown as means ± SD, *n* = 3 biological replicates. In each independent experiment, 15 cells were analyzed. **p < 0.01, ***p < 0.001 versus CT, one-way ANOVA test, calculated with GraphPad Prism 8.

Proper attachment of kinetochore to microtubules is crucial for the accurate segregation of chromosomes. Aurora B plays a critical role in destabilizing aberrant microtubule - kinetochore connections. Aurora B kinase activity is tightly regulated in the process of spindle assembly. Either increased or decreased Aurora B kinase activity disrupts KT-MT attachment and spindle instability. Therefore, we next monitored the impact of BRCC36-KO or Abor1-KO on Aurora B activation using a phospho-specific antibody against the Aurora B activation loop (Aurora B-pT232). As shown in Fig. 3a, the intensity of Aurora B-pT232, but not non-phosphorylated Aurora B, was significantly higher in BRCC36-KO and Abro1-KO cells than in control cells (Fig. 3a, b), indicating that BRISC deficiency promotes Aurora B activation. To further assess Aurora B kinase activity in BRCC36-KO and Abro1-KO cells, we examined the known Aurora B substrates that regulate kinetochore–microtubule (KT-MT) attachments, including Hec1, a substrate at the outer kinetochore, and mitotic centromere-associated kinesin (MCAK), the kinesin-13 microtubule depolymerase implicated in the regulation of KT-MT plus-end dynamics^41^. The intensity of phosphorylated Hec1 (Hec1-pS55) was significantly increased in prometaphase cells when BRCC36 or Abro1 was knocked out, and specific aggregated Hec1-pS55 staining appeared more frequently in BRCC36-KO (74.1%) and Abro1-KO (50%) cells than in that of control cells (12.5%) (Fig. 3c, d), suggesting an increased Aurora B activation in BRISC-deficient cells. Likewise, MCAK was accumulated at the inner centromere, indicated by CREST (human calcinosis, Raynaud’s phenomenon, esophageal dysfunction, sclerodactyly, and telangiectasia) staining, in these KO cells (Fig. 3e, f). We also observed a similar increase in the phosphorylation of histone H3 (H3-pS10), an Aurora B substrate localizing along chromosome arms, in BRCC36-KO and Abro1-KO cells (Supplementary Fig. 2e, f). Together, these results indicate that BRISC deficiency leads to hyperactivation of Aurora B in mitosis, suggesting a role of BRISC in restricting Aurora B kinase activity.

Aurora B activity is critical for establishing proper KT-MT attachment because it regulates the error-correction process and lateral to end-on conversion process during spindle assembly^42,43^. To evaluate the effects of Aurora kinase hyperactivation caused by BRCC36- or Abro1-knockout, we first examined KT-MT attachments by co-staining of α-tubulin and CREST in metaphase cells, synchronized by double thymidine block, followed with MG132 treatment. To visualize the stable microtubules that only attach to kinetochores (K-fibers), cells were subjected to a short cold treatment prior to fixation. End-on stable KT–MT attachments were observed in control cells, whereas some kinetochores were seen not captured by MT in 40-50% BRCC36/Abro1-KO cells, suggesting an unstable attachment between KT and MT in these cells (Fig. 3g, h). Strikingly, majority of the CREST foci were paired and round in metaphase control cells, whereas some CREST foci were not separated as pairs but aggregated or appeared as rod-shaped misshapen in 50% BRCC36/Abro1-KO cells (Fig. 3g, i). To explore the reason that lead to these distort kinetochores, we measured the distance between each paired sister-centromeres. In control cells, bi-oriented end-on KT-MT attachments under full tension had relatively long interkinetochore distance (1.4 ± 0.23 µm), while the interkinetochore distance in KO cells is significantly shortened (1.0 ± 0.26 µm) (Fig. 3j and Supplementary Fig. 3a), suggesting an improper KT-MT connection and reduced tension in KO cells, which leads to defects in paired kinetochores separation and the formation of kinetochores aggregation or rod-shaped misshapen or distortion.

Hyperactivation of Aurora-B is thought to promote lateral attachments^42^. Therefore, we further examined the KT-MT attachment status by immunostaining Astrin, a microtubule-binding protein specifically enriched on end-on kinetochores^42^. As shown in Fig. 3k, l, almost all kinetochores were attached to MT-ends and enriched for Astrin in control cells. However, about 25–30% of BRCC36-KO/Abro1-KO cells showed a significant increase in laterally attachment kinetochores and failed to recruit Astrin to the end of MT but MT walls. Consistently, Mad1 was retained on the lateral attachment kinetochores in BRCC36-KO cells, indicating a spindle assembly checkpoint activation, while it was disappeared in control cells with end-on kinetochores (Supplementary Fig. 3b). These results suggest that BRISC deficiency leads to hyperactivation of Aurora B, which retards the lateral to end-on conversion process and increases/stabilizes lateral attachment that are regulated by Aurora B as well as Astrin-SKAP complex.

Increased Aurora B activity can also lead to continuous disruption of chromosome–microtubule attachments during error-correction process^44^. Thus, we further assessed the error-correction efficiency of Aurora B, using methods to accumulate chromosomes with improper attachments to the spindle, and focusing the analysis on chromosome alignment. First, we treated cells with nocodazole (30 ng/mL) for 4 h to increase the formation of merotelic attachments, a single kinetochore attaches to microtubules from both spindle poles^41,44–46^, then washout and continued to culture for 30 min to allow spindle reassembly, and cells were synchronized to metaphase. We found notable chromosome alignment defects and centromere separation defects in BRCC36/Abro1-KO cells (Supplementary Fig. 3c–e), suggesting a defect in correcting improper chromosome–microtubule attachments in KO cells^41^. Moreover, we treated cells with Monastrol, an Eg5 inhibitor, to arrest mitotic cells with monopolar spindles, in which many chromosomes are mal-orientated, K-fibers attach both sister kinetochores to the same pole (syntelic mal-orientation)^45^. Bipolar spindles and metaphase-aligned chromosomes were formed in control cells after Monastrol removal, while the percentage of misaligned chromosomes was significantly increased in BRCC36/Abro1-KO cells (Supplementary Fig. 3f, g), followed by increased lagging chromosomes in anaphase (Supplementary Fig. 3h, i).

Collectively, these results demonstrate that BRISC is critical for establishing proper KT-MT attachment. Based on the results above, increased Aurora B activity as a result of BRISC knocked out triggers defects in KT-MT attachments, chromosome alignment and chromosome segregation. These phenotypes might be due to: (i) excessive error-correction (hyperactivation of Aurora B continuously phosphorylates its substrates at outer kinetochore, Hec1, promoting detachment of end-on kinetochores and their recapture onto microtubule walls), (ii) excessive stabilization of KT-MT interactions through phosphorylation of MCAK, and (iii) abrogation of the process of converting lateral attachments to end-on attachments via Astrin.

### Ubiquitination at Lys202 is required for Aurora B activation and kinase activity

**Fig. 4:**
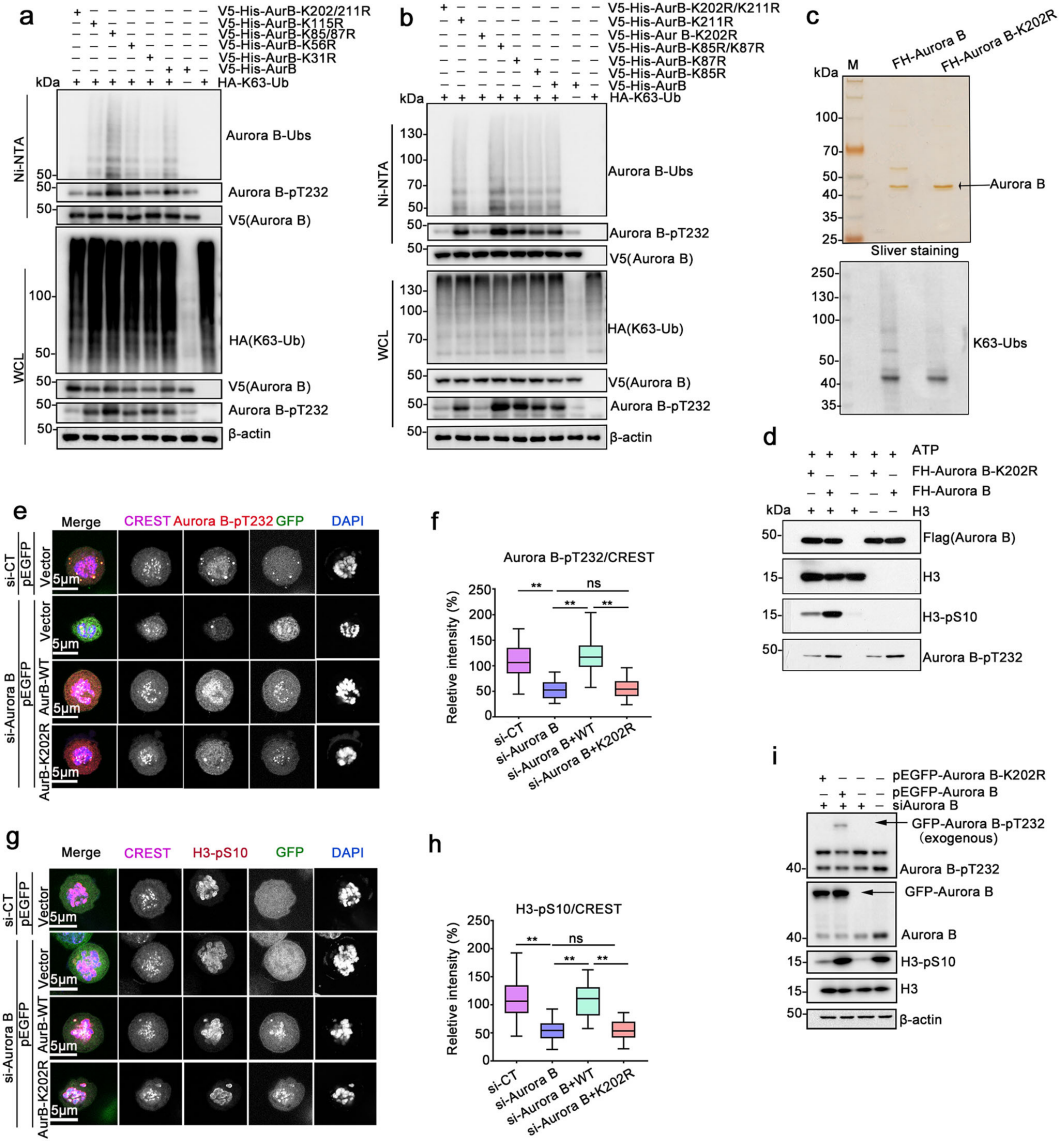
K63-linked ubiquitination at Lys202 is required for Aurora B kinase activity. **a**, **b** K202 is the main ubiquitination site on Aurora B. HEK293T cells were co-transfected with the indicated plasmids and treated with 30 ng/mL NOC for 16 h. Mitotic cells were subjected to Ni-NTA pull-down under denaturing conditions. V5-His-Aurora B pull-downs were analyzed by immunoblotting with the indicated antibodies. **c** Immunoprecipitations used in the in vitro kinase assay. FH-Aurora B or FH-Aurora B-K202R was purified from mitotic HEK293T cells using Flag-M2 agarose beads, resolved by SDS–PAGE and silver staining (upper), and immunoblotted with anti-K63-Ub antibody (bottom). **d** In vitro kinase assay was performed using the purified Aurora B complex shown in **c**, in the presence of ATP. H3 was used as the substrate of Aurora B. **e**–**i** Aurora B-K202R has lower kinase activity in vivo. Representative confocal images showing Aurora B-pT232 (**e**) or H3-pS10 (**g**) intensity in cells transfected with pEGFP-Aurora B or pEGFP-Aurora B-K202R. Endogenous Aurora B was silenced by siAurora B in HeLa cells, followed by co-transfection with the indicated plasmids. Then cells were synchronized with 30 ng/mL NOC, fixed, and stained with the indicated antibodies. Aurora B-pT232, red; CREST, purple; DAPI, blue; H3-pS10, red. Bar, 5 μm. **f**, **h** Box-and-whiskers plots showing the quantification of the relative intensity of Aurora B-pT232 or H3-pS10 as shown in **e** and **g**, respectively. Intensities were normalized to the general kinetochore marker CREST. Boxes show the upper and lower quartiles with a line at the median. Whiskers as the extent of 100% of the data. Data are shown as means ± SD (*n* = 15 cells, and six areas for each cell were counted). **p < 0.01 versus CT, one-way ordinary ANOVA test, analyzed by GraphPad Prism 8. Experiments were repeated at least three times. **i** Protein samples from cells treated as shown in **e** and **g** were analyzed by western blotting. Actin was used as a loading control.

To ascertain the function of K63-linked ubiquitination in regulating Aurora B activity in mitosis, we identified the ubiquitination site(s) on Aurora B. An analysis of the purified Flag–Aurora B protein using mass spectrometry revealed that 7 Lys residues (Lys31, Lys85, Lys87, Lys115, Lys202, and Lys211) of Aurora B might be conjugated to ubiquitin (Supplementary Data 1). Except for K87, the other 6 ubiquitination sites have been assigned in high-through proteomic papers^47–50^. The seven lysine residues were mutated to arginine, co-transfected with HA-K63-Ub in HEK293T cells, and mitotic lysates were subjected to Ni-NTA pull-down. Aurora B K31R shows fewer K63-Ub species and slight weaker Aurora B pT232 as compared to Aurora B WT (Fig. 4a). The K202/211R double mutation almost completely abolished the K63-linked ubiquitination of Aurora B in cells (Fig. 4a); K202R mutation alone also showed a remarkable reduction of K63-Ubs (Fig. 4b). Importantly, consistent with the reduction of K63-linked ubiquitination, phosphorylation of Aurora B (pT232) was decreased either in the whole-cell lysates (WCL) or the His-Aurora B K202R pull-downs (Fig. 4a, b). Besides, amino acid sequences alignment revealed that Lys 202 (K202) is a conserved site in higher mammalian species (Supplementary Fig. 4b), thus we propose that K202 is the primary K63-conjugated ubiquitin site in Aurora B, and K63-linked ubiquitination of Aurora B at K202 could be involved in Aurora B kinase activation.

To verify this, we performed in vitro kinase assay. First, Flag-Aurora B and Flag-Aurora B-K202R were purified from mitotic HEK293T cells, and their ubiquitination status were examined using the K63-Ub antibody. As shown in Fig. 4c (bottom), the K63-Ub species in FH-Aurora B K202R was weaker than that in FH-Aurora B. Next, commercial recombinant histone H3 protein was used as the substrate and incubated with the purified Flag-Aurora B or Flag-Aurora B-K202R for 30 min at 37 °C in the kinase buffer with ATP. As represented by H3-pS10, Aurora B kinase activity was substantially weaker in Aurora B K202R buffer as compared with the wild-type counterparts (Fig. 4d). Notably, the amount of Aurora B-pT232 was less in Aurora B K202R reaction products than in control, suggesting a reduced autophosphorylation ability for Aurora B K202R. These results indicate that the K63-ubiquitination of Aurora B is crucial for its kinase activity.

Furthermore, we examined the intensity of Aurora B-pT232 and H3-pS10 in EGFP-Aurora B K202R-expressing mitotic HeLa cells, in which endogenous Aurora B was depleted by siRNA simultaneously. As shown, the intensity of Aurora B-pT232 was significantly decreased in cells treated with siRNA targeting endogenous Aurora B, while completely restored by re-expression of wild-type EGFP-Aurora B, but not the mutant EGFP-Aurora B K202R (Fig. 4e, f, i). Similar results were obtained for H3-pS10 (Fig. 4g, h, i).

Together, these data demonstrate that K63-Ubs modification at Lys202 is required for Aurora B’s kinase activity.

### Ubiquitination of Aurora B at Lys202 facilitates the formation of CPC complex

**Fig. 5:**
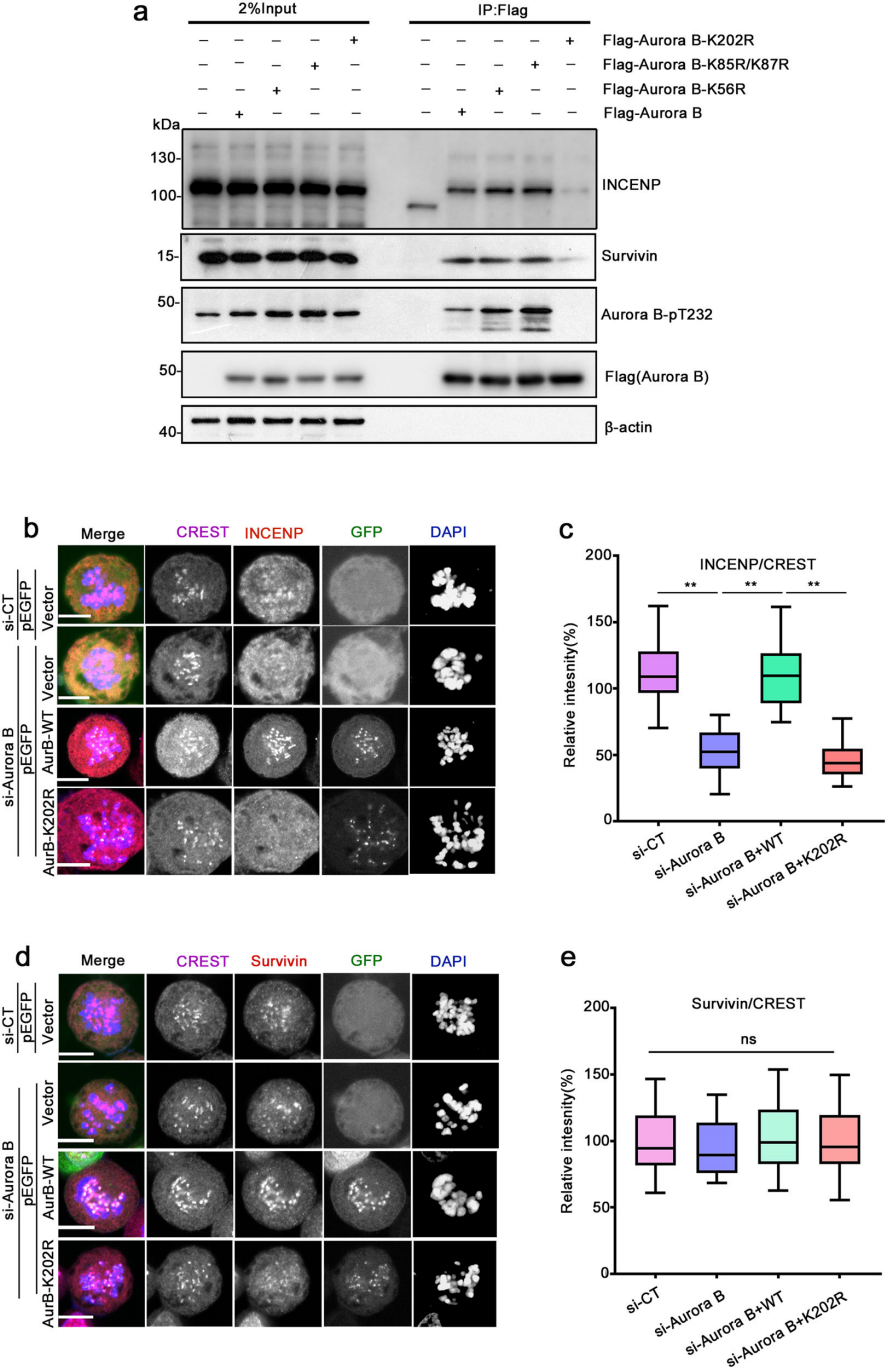
Ubiquitination of Aurora B at Lys202 facilitates the formation of CPC complex. **a** Mutation at Aurora B-K202 disturbs the interaction between Aurora B and INCENP/Survivin. HEK293T cells were transfected with the indicated plasmids and then treated with 30 ng/mL NOC for 16 h, arrested at mitosis, and the lysates were subjected to Flag-IP. The immunoprecipitates were separated and blotted with the indicated antibodies. **b**, **d** Representative confocal images of centromere localization of INCENP (red) (**b**) and Survivin (red) (**d**) in pEGFP-Aurora B or pEGFP-Aurora B-K202R transfected cells. HeLa cells were treated as described in Fig. 4e. Bar, 5 μm. **c**, **e** Box-and-whiskers plots showing the quantification of the relative intensity of INCENP or Survivin shown in **b** or **d**. Intensities were normalized to the general kinetochore marker CREST. Boxes show the upper and lower quartiles with a line at the median. Whiskers as the extent of 100% of the data. *P*-values were calculated by GraphPad Prism 8. Data are shown as means ± SD (*n* = 15 cells, and six areas for each cell were counted). **p < 0.01 versus CT, one-way ordinary ANOVA test.

To understand the precise mechanism by which ubiquitination at K202 promotes Aurora B activation, we first examined the interaction of Aurora B with other members of the CPC complex by Co-IP. Interaction of Aurora B with INCENP or Survivin was almost completely abolished in Flag-Aurora B K202R immunoprecipitates, while no significant alteration was observed in Flag-Aurora B K85R/K87R or Flag-Aurora B K56R immunoprecipitates (Fig. 5a). Notably, the phosphorylation of Aurora B (pT232) was almost undetectable in Flag-Aurora B K202R immunoprecipitates, which is consistent with the in vitro kinase assay (Fig. 4d). Furthermore, we evaluated CPC complex formation by IF in mitotic HeLa cells expressing EGFP-Aurora B K202R while simultaneously knocking down the endogenous Aurora B. The intensity of INCENP was significantly decreased in cells treated with si-Aurora B, while completely restored by re-expression of wild-type EGFP-Aurora B, but not the mutant EGFP-Aurora B K202R (Fig. 5b, c). No significant differences in kinetochore intensities of Survivin were observed between these groups (Fig. 5d, e). These findings suggest that ubiquitination at K202 facilitates the formation of the CPC complex, which in turn benefits the full activation of Aurora B.

### Ubiquitination of Aurora B at Lys202 is required for proper mitotic progression

**Fig. 6:**
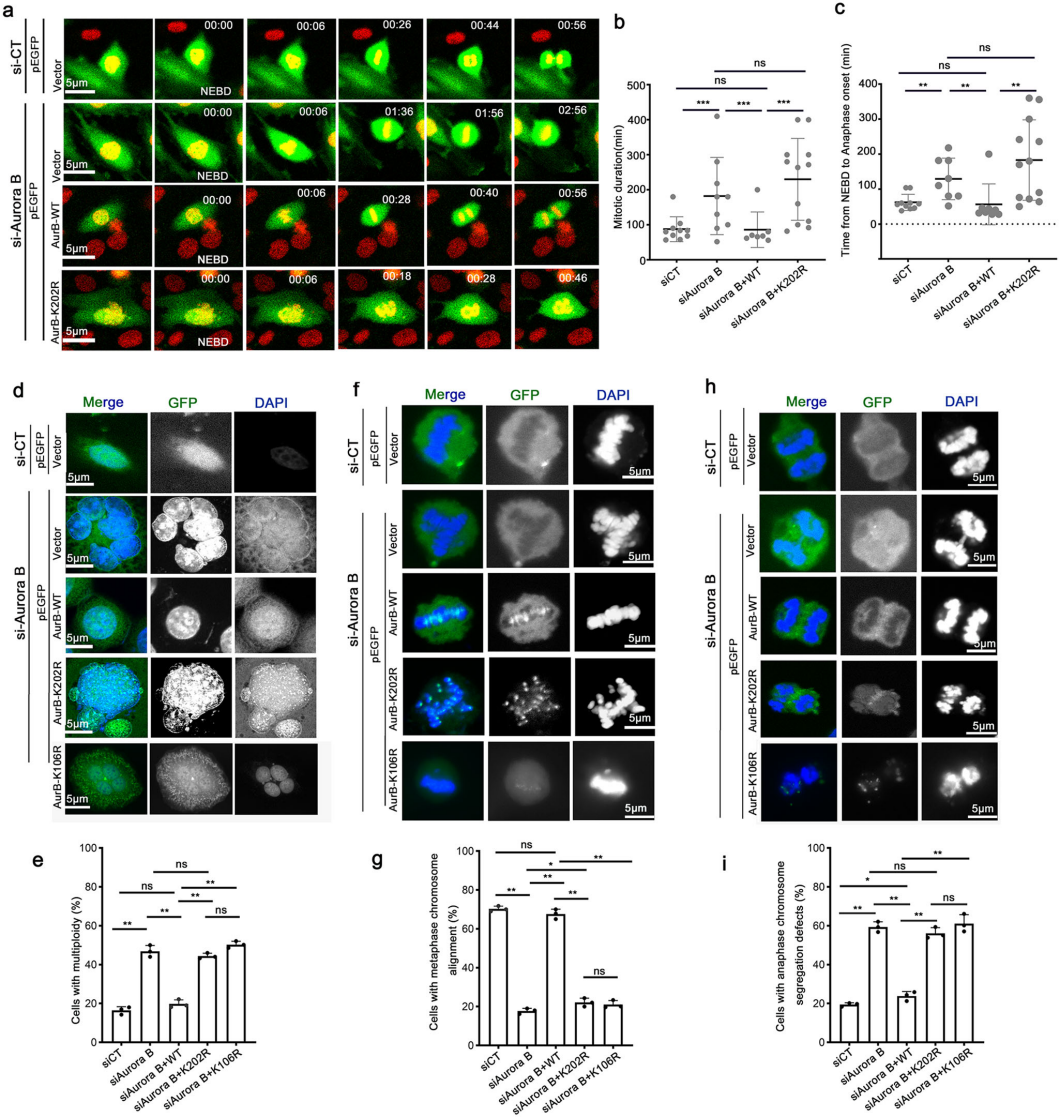
Ubiquitination of Aurora B at Lys202 is required for proper mitotic progression. **a** Representative live-cell still images of dividing mCherry-H2B HeLa cells co-transfected with siAurora B and pEGFP-vector control, pEGFP-Aurora B or pEGFP-Aurora B-K202R, respectively. Bars, 5 µm. NEBD indicates the first frame after nuclear envelope breakdown, based on the chromatin marker mCherry-H2B. Times are shown in hours: min. mCherry-H2B HeLa cells transfected with the indicated plasmids were synchronized by RO-3306 (9 μM) for16 h and then released into a fresh medium. Live-cell confocal time-lapse images were taken at the indicated time points. **b** Quantification of the mitotic duration cells spent from NEBD to mitosis completion. **p < 0.01 **c** Quantification of the time course cells spent from NEBD to anaphase onset. **p < 0.01. Each dot represents an individual cell. **d** Representative images of multi-nuclei defects in post-mitotic cells co-transfected with siAurora B and pEGFP-vector control, pEGFP-Aurora B, pEGFP-Aurora B-K202R or pEGFP-Aurora B-K106R, a known kinase inactive mutant. Bar, 5 μm. **e** Quantification of th**e** cells with multi-nuclei defects shown in **d**. Data are shown as means ± SD, *n* = 3 biological replicates. In each independent experiment, 30 cells were analyzed. **f**–**i** The effects of Aurora B-K202R mutant on KT-MT error correction. Representative confocal images showing metaphase chromosome alignments (**f**) or anaphase chromosomes segregation (**h**) in cells transfected with the indicated plasmids as described in **d**. HeLa cells were synchronized by monastrol (50 μM) and released into a fresh medium for 30 or 45 min. Bar, 5 μm. **g** Quantification of the cells with aligned chromosomes at metaphase shown in **f**. Data are shown as means ± SD, *n* = 3 biological replicates. In each independent experiment, 20 cells were analyzed. **i** Quantification of the cells with chromosome segregation defects at anaphase, shown in **h**. Data are shown as means ± SD, *n* = 3 biological replicates. In each independent experiment, 16 cells were analyzed. **p < 0.01 versus CT, Student’s *t*-test.

To assess whether the K202 ubiquitin site is involved in the function of Aurora B in mitosis, we monitored mitotic progression using live-cell imaging in mCherry-H2B HeLa cells. Cells were depleted of endogenous Aurora B and reintroduced with GFP-Aurora B WT or K202R mutant. The chromosomes, visualized by mCherry-H2B, properly congressed at the metaphase plate and equally divided into the two daughter cells in about 1 h in control cells (Fig. 6a and Supplementary video-1). In contrast, Aurora B-silenced cells took a longer time to align the chromosomes to the equatorial plate, had a prolonged metaphase-like arrest, and took a longer time to complete the mitosis (Fig. 6a–c and Supplementary video-2), consistent with previous reports^4,51^. These mitotic defects caused by depletion of Aurora B could be mostly rescued by restoration of GFP-Aurora B WT, but not the K202R (Fig. 6a–c; Supplementary video 3 & 4). Of those cells with silenced Aurora B, some finally underwent apoptosis due to mitosis catastrophe, while others completed mitosis with multi-nuclei, which was further confirmed with IF assays. As shown (Fig. 6d, e), the percentage of giant multi-nuclei was increased to 50% in Aurora B-silenced cells, indicating a defective cytokinesis. This phenotype caused by Aurora B depletion could be partially rescued by restoration of GFP-Aurora B WT, but not the K202R (Fig. 6d, e). Collectively, these results suggest that ubiquitination of Aurora B at K202 is required for proper mitotic progression.

Next, we tested whether the K202 ubiquitin site of Aurora B is involved in regulating KT-MT attachments using Monastrol treatment as previously described. The percentage of misaligned chromosomes was significantly increased in Aurora B-silenced cells (Fig. 6f, g), followed by lagging chromosomes in anaphase (Fig. 6h, i), which could be rescued by restoration of GFP-Aurora B WT, but not the K202R. Together, these results suggest that the K202 ubiquitin site is crucial for the function of Aurora B in mitosis.

In addition, we observed similar phenotypes when restored GFP- Aurora B K106R, a known catalytically inactive Aurora B mutant^20^, to the Aurora B-silenced cells, as compared with the restoration of GFP-Aurora B K202R (Fig. 6d–i). This further support that the K202 ubiquitin site is crucial for the activation and mitotic function of Aurora B.

### Ubiquitination of Aurora B at Lys202 is required for cell survival

**Fig. 7:**
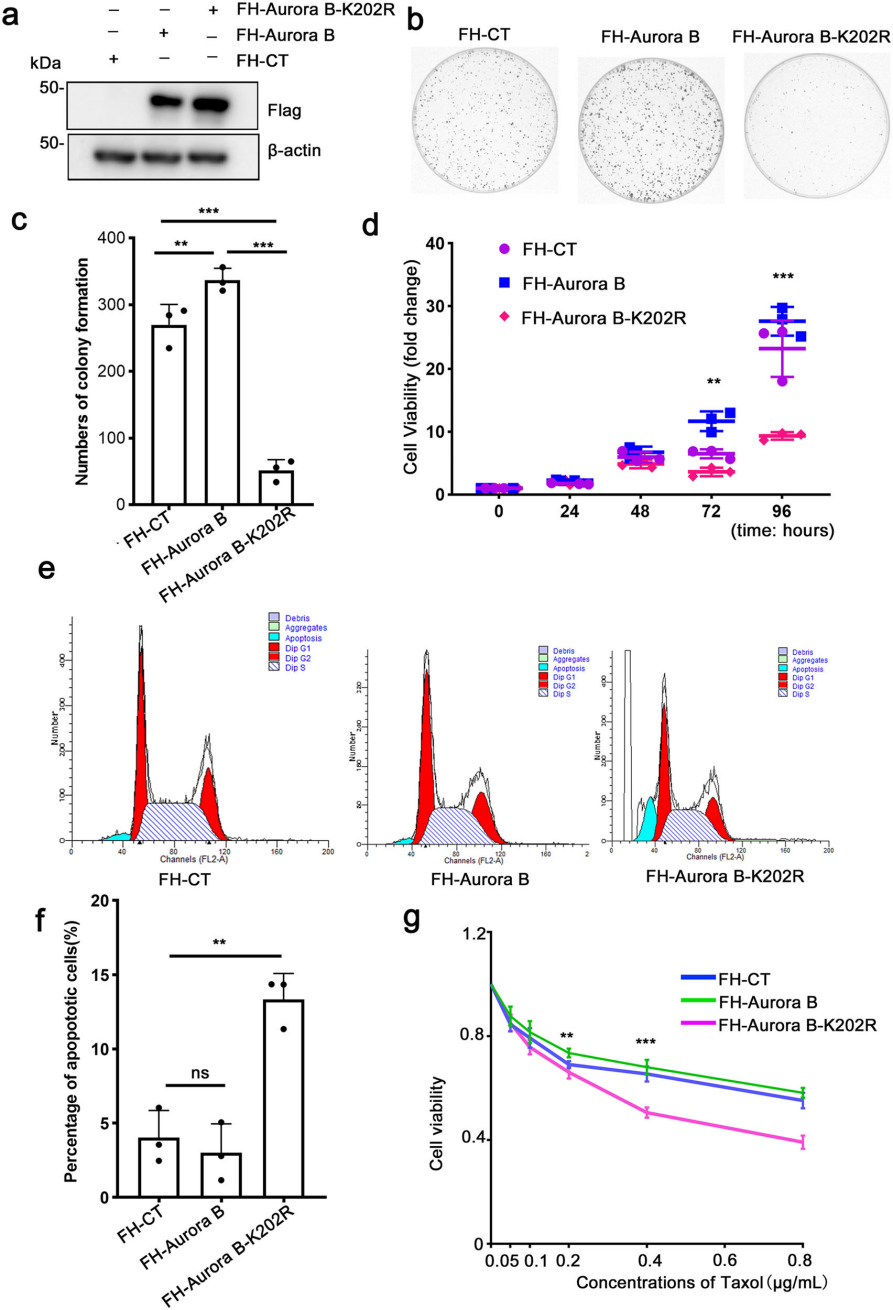
Ubiquitination of Aurora B at Lys202 is required for cell survival. **a** Verification of HeLa cells that stably expressing FH-Aurora B or FH-Aurora B-K202R. Protein samples from the indicated cells were separated by SDS–PAGE, followed by IB. **b** Representative images of colonies formed with the indicated stable cell lines. **c** Quantification of the colony numbers formed in **b**. Data are shown as means ± SD of three independent experiments. **p < 0.01, ***p < 0.001 versus CT, Student’s *t*-test. **d** Effects of Aurora B-K202R on cell viability. CCK8 assay was performed with stable cell lines as described in **a**. Cell viability was normalized to the start time point. Data are shown as means ± SD of three independent experiments. **p < 0.01, ***p < 0.001 versus CT, Student’s *t*-test. **e** Flow cytometry assay was conducted to evaluate the effect of Aurora B-K202R on apoptosis. **f** Quantification of the apoptotic cells shown in **e**. Data are shown as means ± SD of three indep**e**ndent experiments. **p < 0.01, versus CT, Student’s *t*-test. **g** Aurora B-K202R confer sensitivity to the anti-tumor drug, taxol. CCK8 assay was performed with the indicated stable cell lines and treated with taxol for 48 h at the indicated concentrations. Data are shown as means ± SD of three independent experiments. **p < 0.01, ***p < 0.001 versus CT, Student’s *t*-test.

Since aneuploidy is a hallmark of tumors, we next investigated the role of Aurora B K202 ubiquitination in cancer cell proliferation and survival. First, we generated cell lines that stably expressing FH-Aurora B or FH-Aurora B K202R (Fig. 7a), then the effect of FH-Aurora B K202R on colony formation was tested in these cell lines. The capacity of colony formation was dramatically reduced in FH-Aurora B K202R-expressing cells compared to FH-Aurora B-expressing and control cells (Fig. 7b). The colony number formed by cells stably expressing with Fag-HA-Aurora B was higher than control cells (Fig. 7b, c), which is in accordance with previous reports that Aurora B overexpression enhances cancer cells proliferation^9,52,53^. Furthermore, the CCK8 assay was used to test cell viability. The viability was increased in FH-Aurora B-expressing HeLa cells, while significantly decreased in FH-Aurora B K202R-expressing HeLa cells, as compared with control cells (Fig. 7d). In addition, we observed the effect of FH-Aurora B K202R on apoptosis. The percentage of subG1 (apoptotic cells) was increased to 13.33% in cells expressing FH-Aurora B-K202R compared with control (4.02%) or wild-type cells (3.01%) (Figs. 7e, f and Supplementary Fig. 5).

Previous studies have shown that inhibition of Aurora B could confer sensitivity to Taxol in HeLa cells^54–56^. To test this, HeLa cells stably expressing FH-Aurora B or FH-Aurora B-K202R were exposed to Taxol treatment for 48 h at the indicated concentration, and then tested by CCK8 kit. The cell viability was dramatically decreased in FH-Aurora B-K202R-expressing cells compared to FH-Aurora B-expressing and control cells (Fig. 7g). Together, these findings showed that Lys202 of Aurora B is a critical site for cell survival.

## Discussion

Aurora B is the enzymatic core of the CPC and is an essential regulator for the progression of mitosis. Its activation has primarily been investigated in the context of protein phosphorylation. Here, we identify a mechanism by which K63-linked polyubiquitination regulates Aurora B activation in mitosis and identify BRISC as an important regulator of Aurora B.

**Fig. 8:**
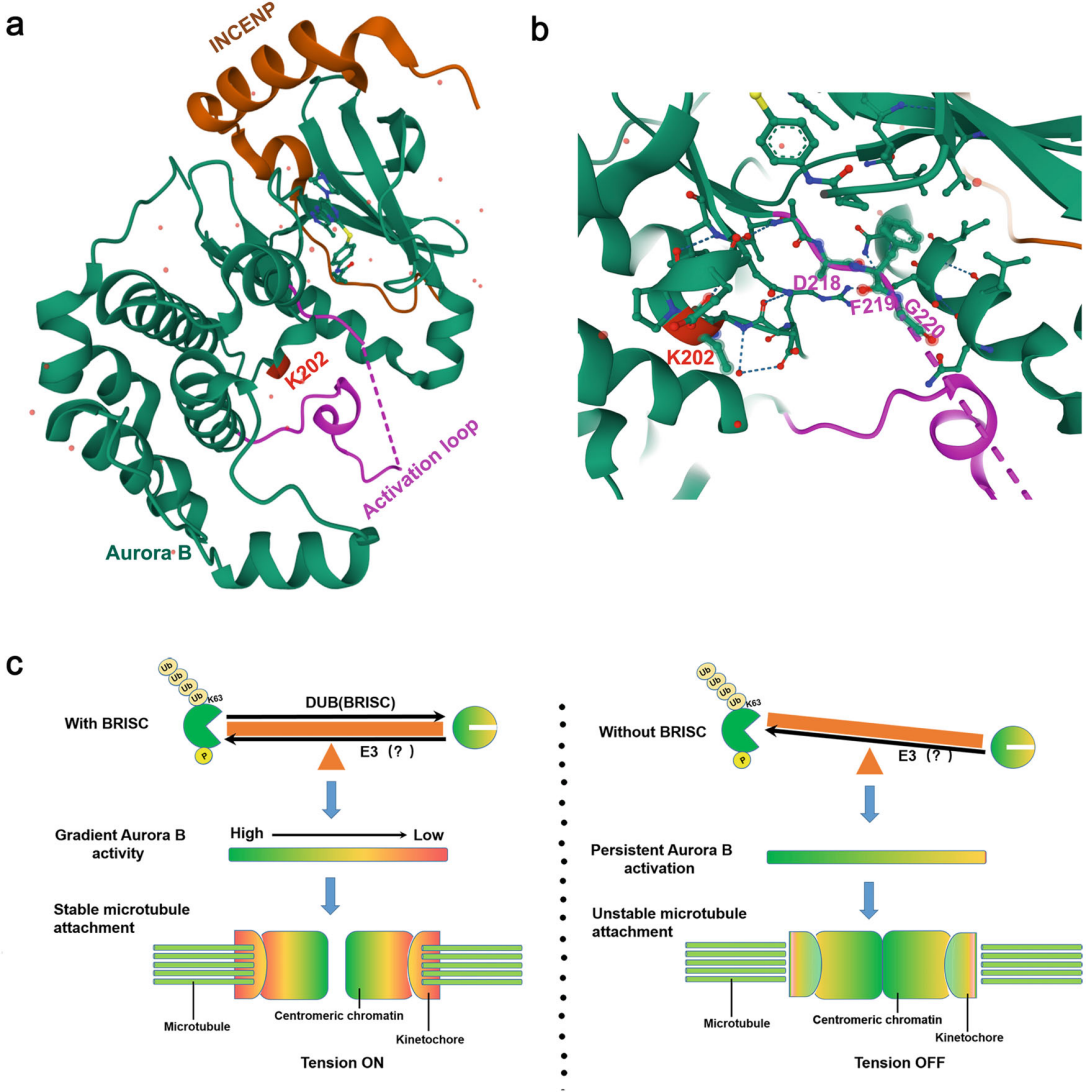
A working model explaining the mechanism by which Aurora B activation and kinase activity are regulated by K63-lined ubiquitination. **a** The crystal structure of human Aurora B kinase in complex with INCENP and VX-680 (PDB ID: 4AF3)^58^. K202 (red) is located in the activation loop (purple) of Aurora B (green). **b** Magnified view showing the position of K202 and surrounding DFG (Asp-Phe-Gly) motif at the start of the activation loop. The crystal structure of human Aurora B Kinase provides us with strong support. We propose that ubiquitination at K202 might be critical for the autoactivation of Aurora B, which in turn facilitates the phosphorylation of TSS in INCENP, required for CPC complex formation and full activation of Aurora B. **c** Schematic showing BRISC-regulated K63-Ubs of Aurora B is important for creating a dynamic gradient Aurora B kinase activity to ensure correct KT-MT attachment and chromosome segregation.

APC/C-mediated K11-linked ubiquitination has a critical role in targeting Aurora B for degradation. However, protein-degradation-independent signaling is also involved in its function in mitosis. In this study, we reveal that K63-linked ubiquitination is required for Aurora B activity and function in mitosis. Our data demonstrate that Aurora B is modified by K63-linked polyubiquitination chains during mitosis, K202 is the primary K63-conjugated ubiquitin site in Aurora B. K63 ubiquitination of Aurora B facilitates Aurora B activity, mutating of Aurora B at K202 disturbs its interaction with INCENP and Survivin, leading to reduced activation of Aurora B, which is deleterious for MT-KT attachments and for mitotic progression. Although the precise mechanism of how Aurora B K63-Ubs facilitates its activation has yet to be fully established, other studies on protein crystallography provide us with reasonable support. K202 is located in the activation groove of Aurora B and surrounded by the DFG (Asp-Phe-Gly) motif at the start of the activation loop^57,58^ (Fig. 8a, b). Hence, one possibility is that ubiquitination at this site might be critical for the autoactivation of Aurora B^58^. This in turn facilitates the phosphorylation of TSS in INCENP, which is important for CPC complex formation and full activation of Aurora B. Future studies will be needed to test this hypothesis.

In addition, we find that BRISC is the DUB responsible for removing K63-Ubs from Aurora B, and is important for restricting Aurora B activation. Aurora B partially co-localizes with BRCC36 or Abro1 throughout mitotic stages. Depletion of BRISC enhances the accumulation of K63-Ub chains for Aurora B, resulting in its persistent activation, leading to erroneous accumulation of chromosome–microtubule attachments and severe mitotic defects.

Based on our results, both increased and decreased Aurora B ubiquitination that were caused, respectively, by BRISC knocked out or K202 mutation (K202R) could lead to defects in KT-MT attachments and mitotic progression, indicating that the dynamic K63-ubiquitination and deubiquitylation process of Aurora B is important for Aurora B’s activity and function in mitosis. Aurora B functions to correct aberrant kinetochore–microtubule interactions by altering the activity of kinetochore components, including NDC80 (Hec1). Aurora B activity is high on misaligned kinetochores that are not under tension, but is reduced at aligned kinetochores. Active Aurora B lost from outer kinetochore and retained only at the inner centromeric region is essential for establishing tension-on KT-MT attachments during metaphase. Our findings suggest that timely removal of K63-Ubs from Aurora B by BRISC facilitates Aurora B inactivation at aligned kinetochores. Continuous Aurora-B activity due to depletion of BRISC promotes the incidence of lateral kinetochores, leading to mitotic defects. Thus, we propose that balanced K63-linked ubiquitination and deubiquitination of Aurora B are necessary for generating a spatial gradient of Aurora B activity at the centromere and KT-MT interface through mitosis, which is critical for establishing stable KT-MT attachments and bi-oriented chromosomes at metaphase (Fig. 8c).

Ubiquitination is a dynamic reversible process that is regulated by multiple E3 ligases and DUBs. Further studies addressing the role of these potential K63-E3 ligases regulating Aurora B will be essential for understanding the mechanism of the K63-linked ubiquitination in its mitotic function.

Aurora B is overexpressed in many human tumors and becomes to be a promising target in cancer therapy. Several Aurora B inhibitors are undergoing preclinical validation, but the dose-dependent toxicity and the resistance mediated by single-site mutations in the active site of Aurora B remain major concerns that limit their development^57^. We present here that mutation at K202 reduces Aurora B kinase activity, promotes cell apoptosis, and enhances the efficiency of the anti-tumor drug Taxol. Thus, our findings provide a potential site for Aurora B-targeting drug design, which might improve the sensitivity of Aurora B kinase inhibitors.

## Methods

### Plasmid construction

Full-length cDNA encoding Aurora B was amplified by PCR with Phusion DNA polymerase (TOYOBO, Inc.) and subcloned into the plasmids pcDNA3.1-V5-His-B, pEGFP-C2 (CMV promoter; Takara Bio Inc.), pcDNA3.1-Flag-HA (CMV promoter) or pGEX-6P-2 plasmid (tac promoter; GE Healthcare) to generate pcDNA3.1-V5-His-Aurora B, pEGFP-Aurora B, pcDNA3.1-HA-Aurora B, and pGEX-6P-2-Aurora B. Aurora B mutants were generated using site-directed mutagenesis. For generation of stable HeLa BRCC36 or Abro1 knock out cells, sgBRCC36/sgAbro1 (designed on https://chopchop.cbu.uib.no) were subcloned into the lentiCRISPRv2 vector using the following oligonucleotides: sgBRCC36-1-Forward-5′-CACCGAAGTAATGGGGCTGTGCATA-3′; sgBRCC36-1-Reverse-5’-AAACTATGCACAGCCCCATTACTTC-3’; sgBRCC36-2-Forward-5′CACCGGAGCGTGGTTGAGACAAACG-3’; sgBRCC36-2-Reverse-5′- AAACCGTTTGTCTCAACCACGCTCC-3’; sgAbro1-1-Forward-5′- CACCGGTCGGGCACGCCGAGGATGC-3’; sgAbro1-1-Reverse-5′- AAACGCATCCTCGGCGTGCCCGACC-3’; sgAbro1-2-Forward-5′-CACCGGCGAGTTGTTGAATCTTGTCAGG-3’; sgAbro1-2-Reverse-5′- AAACCCTGACAAGATTCAACAACTCGCC. sgRNA-resistant plasmids pEYFP-BRCC36res WT or pEYFP-BRCC36res QSQ were constructed by site-directed mutagenesis using pEYFP-C1-BRCC36 WT/QSQ plasmids as templates, and the following oligonucleotides as primers:

Rescue-forward-5’-GCGATCGCCGGCGCGCCATGGACTACAA GGACGACGATGACAAGGGCGGCATGGCCGTCCAAGTGGTGCAG-3’; Rescue-reverse-5’- TGAGTTTCTGCTCGAGTTATTCTAGAGAAGAAAGTTCTTGCATAAGC-3’. Aurora B siRNA sequences were as: 5’- GGAUCCCUAACUGUUCCCU-3’ and 5’- AACGCGGCACUUCACAAUUGA-3’.

### Cell culture and transfection

Human cervical carcinoma HeLa and human embryo kidney HEK293T cells, purchased from American Type Culture Collection and kept in our lab, were cultured in DMEM (Macgene) with 10% FBS (Hyclone) supplemented with 100 µg/ mL penicillin & streptomycin in a 37 °C incubator with a humidified 5% CO_2_ atmosphere. Transfection was performed using jetPRIME® (Thermofisher) according to the manufacturer’s instructions.

### Cell cycle synchronization

For mitotic cells used in IP: exponential cells were synchronized by a standard double thymidine block or by 30 ng/mL nocodazole (Sigma-Aldrich) treatment for 16 h, collected by mitotic shake-off. For mitotic cells used in IF: cells were treated with 30 ng/mL nocodazole for 4 h or with 9 μM RO3306 (Selleck) for 16 h, and released into a fresh medium for the indicated period. For monopolar mitotic cells: cells were treated with 50 μM Monastrol (Selleck) for 4 h, then released into a fresh medium and cultured for the indicated period.

### Establishment of BRCC36 or Abro1 knockout stable cell line

The lentiCRISPRv2 lentiviral vector was used to express the sgRNA that targeting the gene interested. Lentiviral particles were produced and harvested in HEK293T cells. HeLa cells were infected with the lentiviral particles and subjected to puromycin (10 μM) (Selleck) selection to obtain stably expressing cell lines as previously described^59^.

### Co-immunoprecipitation assay

Mitotic cells were harvested and lysed in NETN 400 buffer (400 mM NaCl, 20 mM Tris-Cl (pH 7.4), 0.1% Nonidet P-40, 0.5 mM EDTA, 1.5 mM MgCl_2,_ and 10% Glycerol) with protease inhibitors (P8340, Sigma-Aldrich) and protein phosphatase inhibitors (Selleck) on ice for 30 min. The samples were centrifuged at 12,000 rpm for 15 min, and the supernatants were diluted with NETN-0 buffer, the NETN 400 buffer without NaCl, to obtain a final concentration of NaCl at 150 mM. For endogenous immunoprecipitation, the diluted lysates were incubated with 0.27 μg Aurora B antibody (CST, 3094S) or 2 μg Abro1 antibody^28^ or normal rabbit IgG at 4 °C with rocking for 12 h, then Protein G beads were added, and the incubation was continued for an additional 4 h. For Flag-immunoprecipitation, protein samples and anti-Flag-M2 agarose beads (Sigma-Aldrich) were incubated at 4 °C with rocking for 4-6 h. Agarose beads were then washed three times using the NETN-150 buffer. The bound proteins were eluted with 100 mM glycine, pH 2.5, and then neutralized by adding 1/10 vol of 1 M Tris-Cl, pH 8.0. Eluted proteins were separated on 4–12% SDS–PAGE and blotted with the corresponding antibodies as indicated.

### Ni-NTA pull-down assay

HEK293T cells were seeded in a 6-well plate, transfected with the indicated plasmids, and synchronized to mitosis with 30 ng/mL NOC. Mitotic cells were harvested and lysed in Urea buffer B (8 M urea, 100 mM NaH_2_PO4, 10 mM Tris-Cl (pH 8.0), and 25 mM imidazole, supplemented with protease inhibitors (P8340) and protein phosphatase inhibitors (Selleck)) on ice for 10 min, then subjected to ultrasonic cracking for 30 s (MW 30 W). The whole-cell lysates (WCL) were centrifuged at 12,000 rpm for 15 min. Proteins were precipitated with Ni-NTA agarose for 4 h, followed by rinsing four times with Urea buffer C (8 M urea, 100 mM NaH_2_PO4, 10 mM Tris-Cl (pH 6.3), 25 mM imidazole). The precipitated proteins were eluted with Urea buffer E (8 M Urea, 100 mM NaH_2_PO4, 10 mM Tris-Cl (pH 4.0), 250 mM imidazole) and separated by SDS–PAGE, and analyzed by immunoblotting with the indicated antibodies.

### GST pull-down assay

Recombinant protein GST-Aurora B or GST protein control was expressed in *E.coli* Rosetta, induced with 1 μM IPTG (Sigma-Aldrich) at 16 °C for 16 h. The bacteria were collected and lysed in PBS with PMSF (Sigma-Aldrich) and 25 μg/mL lysozyme (Sigma-Aldrich), then subjected to ultrasonication for 120 s (MW 50 W). GST-fused proteins were immobilized on glutathione sepharose at 4 °C for 4 h. Mitotic HeLa cell extracts were incubated with the immobilized GST-fusion proteins at 4 °C for 4 h. The bound proteins were separated by SDS–PAGE and blotted with the indicated antibodies.

### Immunofluorescence microscopy

Cells grown on coverslips pre-coated with 0.1 mg/mL Poly-l-Lysine (Beyotime) were fixed in cold methanol on ice for 3 min, then fixed with 4% paraformaldehyde in PHEM buffer (60 mM PIPES, 25 mM HEPES, pH 6.9, 10 mM EGTA, 4 mM MgSO_4_) for 10 min, extracted with 0.5% Triton X-100 in PHEM buffer for 15 min, and blocked by 10% goat serum at 37 °C for 30 min, followed by incubation with primary antibody at 4 °C for 14 h, rinsed by PBS supplemented with 0.1% Triton-X-100 for three times, and probed with a secondary antibody at 37 °C for 30 min, washed extensively and incubated with 1 μg/mL DAPI (Pharma biology) in dark for 10 min. Coverslips were mounted with H1700 (Invitrogen). Fluorescence images were captured by a laser confocal microscope (LSM880, Zeiss; TCS-SP8 DIVE, Lecia) and a 63×, 1.40 NA oil objective. Images were processed using Adobe Photoshop CC2018.

### Live-cell imaging

Transfected mCherry-H2B HeLa cells were seeded onto Petri dishes with a 15-mm glass base (NEST), grown in DMEM media supplemented with 10% FBS, and treated with RO3306 (Selleck) to arrest in the G2 phase. Fluorescence time-lapse images were taken every 5 min for 24 h at 37 °C in 5% CO2 by using a laser-scanning confocal microscope (LSM880, Zeiss), with a 10×, 1.4-NA objective. Adobe Photoshop CC2018 was used for image processing.

### In vitro H3 phosphorylation assay

Purified Flag-Aurora B or Flag-Aurora B-K202R protein from HEK293T cells were incubated with 0.5 μg Human Recombinant H3 (NEW ENGLAND BioLabs) for 30 min at 37 °C in the kinase buffer (25 mM Tris-Cl pH7.5, 5 mM β-glycerophosphate, 2 mM DTT, 0.1 mM Na3VO4, 10 mM MgCl_2_, 1 mM ATP). The reaction was terminated by adding SDS sample buffer and subjected to SDS–PAGE.

### CCK-8 assay

A CCK-8 kit (Beyotime) was used to evaluate the survival of HeLa cells stably expressing Flag-HA-Aurora B or Flag-HA-Aurora B-K202R. Cells were seeded at a density of 1000 cells per well in a 96-well plate in five replicates, and cultured for the indicated period. Absorbance was detected using a microplate reader (Tecan).

### Colony formation assay

HeLa cells stably expressing Flag-HA-Aurora B or Flag-HA-Aurora B-K202R were seeded at a concentration of 3000 cells in 35 mm dishes and incubated at 37 °C with 5% CO2 for 14 days. Cells were fixed with 4% paraformaldehyde at room temperature for 30 min and stained with 0.1% crystal violet for 5 min.

### Statistics and reproducibility

All data are shown as mean ± standard deviation. Results were compared by Student’s *t*-tests or one-way ANOVA. Statistical calculations were performed using ZEN 3.1 and GraphPad Prism 8.0. *p*-values < 0.05 is considered statistically significant.

### Reporting summary

Further information on research design is available in the Nature Portfolio Reporting Summary linked to this article.

## Supplementary information

Supplementary Information

Description of Additional Supplementary Files

Supplementary Data 1

Supplementary Video 1

Supplementary Video 2

Supplementary Video 3

Supplementary Video 4

Reporting Summary

## Data Availability

All data generated or analyzed during this study are included in this published article and its supplementary information files. Uncropped scans of blots were provided as Supplementary Figs. 6–17. The raw data based on LC-MS/MS were deposited at Figshare with a 10.6084/m9.figshare.21541524. The Supplementary videos 1–4 related to Fig. 6a were deposited at Figshare with a 10.6084/m9.figshare.21561786. Antibodies used in this study were listed in Supplementary Tables S1 & S2.
