# Supplementary material for: The deubiquitinating enzyme complex BRISC regulates Aurora B activation via lysine-63-linked ubiquitination in mitosis
Source: Commun Biol. 2022 Dec 6;5:1335. doi: 10.1038/s42003-022-04299-4 (PMC9726926; doi:10.1038/s42003-022-04299-4)
Supplement: Supplementary file 1 — Supplementary Information [file 42003_2022_4299_MOESM1_ESM.pdf]

Title: The deubiquitinating enzyme complex BRISC regulates Aurora B activation via lysine-63-linked ubiquitination in mitosis

Supplementary Figure

Supplementary Figure 1. Aurora B interacts with Abro1 and BRCC36 in mitosis. Related to Fig. 2.

Supplementary Figure 2. BRISC deficiency increases Aurora B activity but does not affect Aurora B stability or centromere localization. Related to Fig.3.

Supplementary Figure 3. BRISC deficiency causes erroneous kinetochore-microtubule attachments and abnormal chromosome segregation. Related to Fig. 3.

Supplementary Figure 4. Lys202 is the potential ubiquitination site of Aurora B. Related to Fig. 4.

Supplementary Figure 5. A figure exemplifying the gating strategy used in Flow Cytometry that is related to Fig. 7e.

Supplementary Figure 6 - Supplementary Figure 17. Uncropped scans of western blots for Fig.1, Fig.2, Fig.4, Fig. 5, Fig. 7, and FigS. 1.

Supplementary Table

Supplementary Table S1. List of primary antibodies used in this study.

Supplementary Table S2. List of secondary antibodies used for immunofluorescent staining in this study.

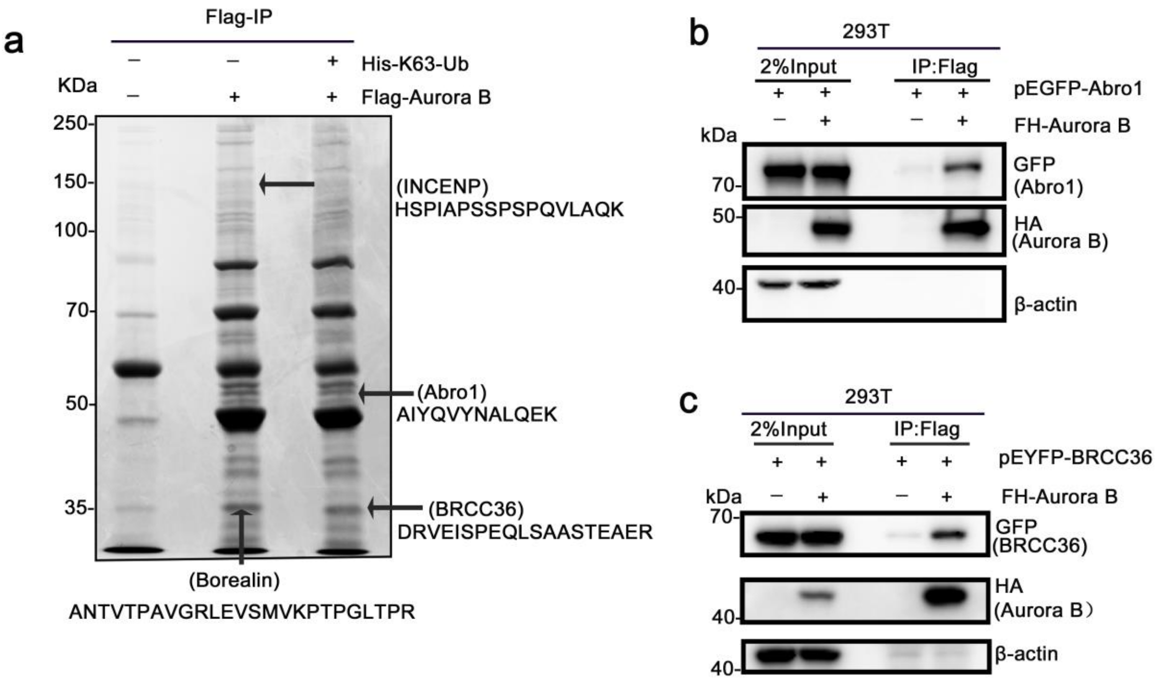

Supplementary Figure 1. Aurora B interacts with Abro1 and BRCC36 in mitosis. Related to Fig. 2. a Immunoprecipitates from mitotic HEK293T cells co-transfected with the indicated plasmids by using anti-FLAG M2 agarose beads were separated by SDS-PAGE, stained with Coomassie Blue, and subjected to mass spectrometry analysis. **b, c** Flag-HA-Aurora B (FH-Aurora B) interacts with Abro1 (**b**) or BRCC36 (**c**) ectopically expressed in HEK293T cells. Mitotic cells co-transfected with FH-Aurora B and pEGFP-Abro1 or pEYFP-BRCC36 were harvested and subjected to immunoprecipitations using anti–FLAG-M2 agarose beads, followed by IB with indicated antibodies.

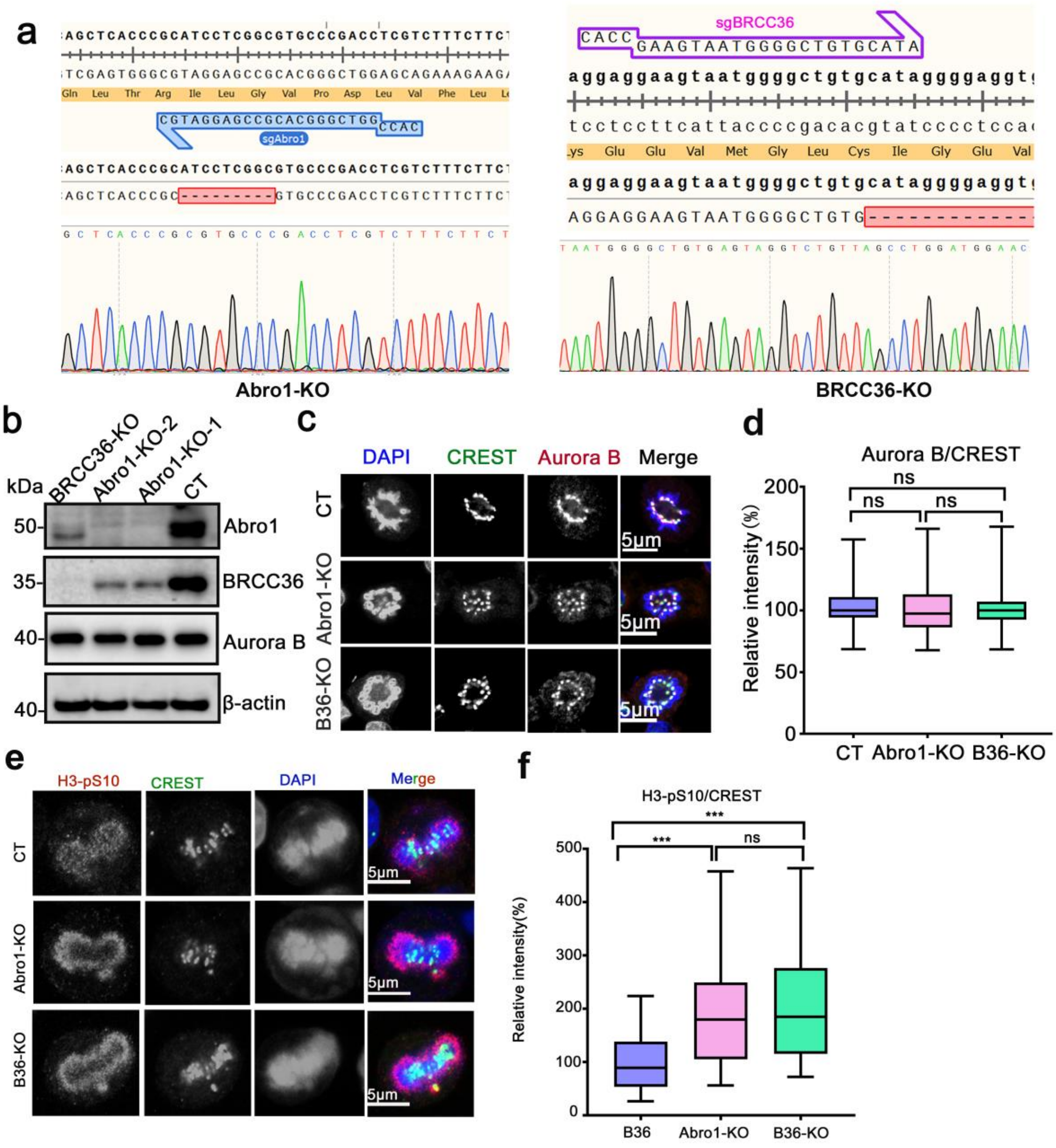

Supplementary Figure 2. BRISC deficiency increases Aurora B activity but does not affect Aurora B stability or centromere localization. Related to Fig.3. a Upper: the sgRNA sequence used for the generation of Abro1-knockout (Abro1-KO, left panel) or BRCC36-knockout (BRCC36-KO, right panel) cell lines. Lower: the genome sequencing results from the stable Abro1-KO (left panel) or BRCC36-KO (right panel) cell lines used in this study. **b** Knockout efficiency of the stable BRCC36-KO and Abro1-KO cell lines was confirmed by Western blotting using the indicated antibodies. **c, d** Protein level of Aurora B was not affected in

BRCC36-KO and Abro1-KO cells. **c** Representative immunofluorescence images of the intensity of Aurora B (red) and CREST (green) in BRCC36-KO or Abro1-KO HeLa cells. DNA was stained with DAPI (blue). Bar, 5 μm. **d** Quantification of the relative Aurora B/CREST intensity shown in **c**. Data are shown as means ± SD (n=15 cells and six areas in each cell were counted). ns, no significance. One-way ordinary ANOVA test. Experiments were repeated three times. **e** Representative confocal images showing Aurora B catalytic activity on its substrate histone H3 in BRCC36-KO or Abro1-KO HeLa cells.Cells were treated with 30ng/mL nocodazole for 4 h and released into fresh medium for 30 minutes, fixed, and stained with indicated antibodies. H3-pS10, red; CREST, green; DAPI, blue. Bar, 5 μm. **f** Quantification of the relative intensities about H3-pS10, normalized to the general kinetochore marker CREST, shown in **e**. Data are shown as means ± SD. ****p*<0.001 versus CT, one-way ordinary ANOVA test, calculated with GraphPad Prism 8. Experiments were repeated three times.

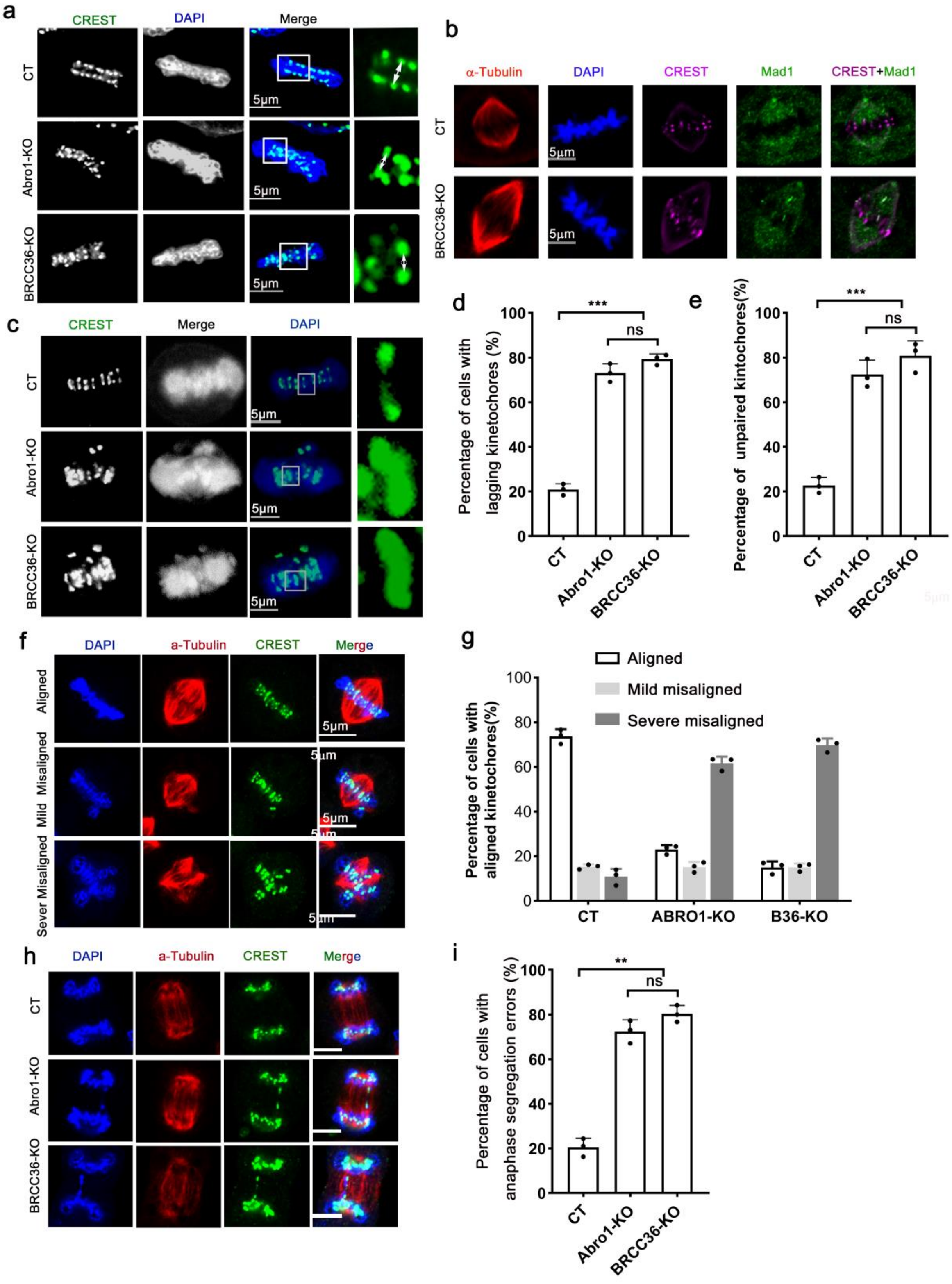

Supplementary Figure 3. BRISC deficiency causes erroneous kinetochore-microtubule attachments and abnormal chromosome segregation. Related to Fig. 3. a Representative confocal images

showing the interkinetochore distance in BRCC36-KO or Abro1-KO HeLa cells. Cells were treated with 10 μM MG132 for 4 h, then fixed, and stained for the kinetochore marker CREST (green). The white arrows indicate paired kinetochores. Bar,5 μm. **b** Representative confocal images showing spindle assembly checkpoint activated, indicated by Mad1, in BRCC36-KO cells with abnormal KT-MT attachments. HeLa cells were treated with a double thymidine block, then released for 6h, followed by 10μM MG132 treatment for 4h to prevent cells from metaphase to anaphase transition. Cells were fixed on ice to destabilize non-kinetochore microtubules and stained for α-tubulin (red), Mad1 (green), CREST (purple), and DNA (DAPI, blue). Bar, 5 μm. **c** Representative confocal images of paired kinetochores in BRCC36-KO or Abro1-KO cells. Cells were treated with 30 ng/mL NOC and released into a fresh medium for 30 minutes, then fixed and stained for CREST (green) and DNA. Bar, 5 μm. **d, e** Quantification of the cells with unaligned kinetochores (**d**) or distorted kinetochores (**e**) shown in **c**. Data are shown as means ± SD from 3 biological replicates. In each independent experiment, 35 cells were analyzed. n.s., no significance. ***p<0.001 versus CT. Student's *t* test. **f** Representative confocal images showing chromosome alignments at metaphase in Abro1-KO or BRCC36-KO cells. HeLa cells were synchronized by monastrol (50 μM) and released into a fresh medium for 30 minutes. 'Aligned', 'Mild misaligned', or 'Severe misaligned' indicate cells with 0, 1–5,or more than 5 unaligned kinetochores in metaphase, respectively. Bar, 5μm. **g** Quantification of the cells with unaligned chromosomes shown in **f**. Data are shown as means ± SD from 3 biological replicates. In each independent experiment, 66 cells were analyzed. **h** Representative confocal images showing lagging chromosomes at anaphase in Abro1-KO or BRCC36-KO cells. **i** Quantification of the cells with chromosome segregation defects shown in **h**. **p<0.01 versus CT, Student's *t* test, Data are shown as means ± SD from 3 biological replicates. In each

independent experiment, 45 cells were analyzed.

a

Site	Sequence	# PSMs	Modification	MH+ [Da]	ΔM [ppm]	m/z [Da]
K202	DIkPENLLLGLK	1	K3(GG)	1466.862	3.067435	489.626
K211	DIKPENLLLGLkGELK	1	K12(GG)	1894.108	4.015253	632.041
K87	GkFGNVYLAR	1	K2(GG)	1238.668	3.37636	619.838
K85	HFTIDDFEIGRPLGkGK	2	K15(GG)	2044.074	6.165568	511.774
K31	kEPVTPSALVLMSR	2	K1(GG)	1641.909	5.717284	547.974
K56	SNVQPTAAPGQkVMENSSGTP	1	K12(GG)	2812.399	3.462101	938.138
K115	SQIEkEGVEHQLR	1	K5(GG)	1666.857	3.788545	833.932

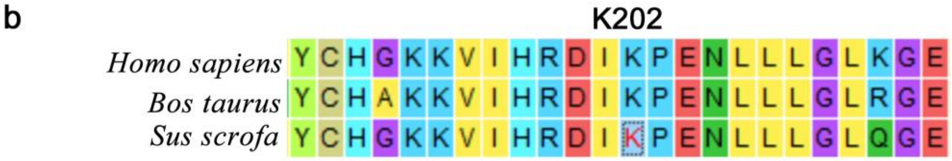

Supplementary Figure 4. Lys202 is the potential ubiquitination site of Aurora B. Related to Fig. 4. a The potential K63-linked ubiquitination sites of Aurora B identified by mass spectrum analysis. **b** Alignment of Aurora B sequences around the K202 ubiquitination sites among species.

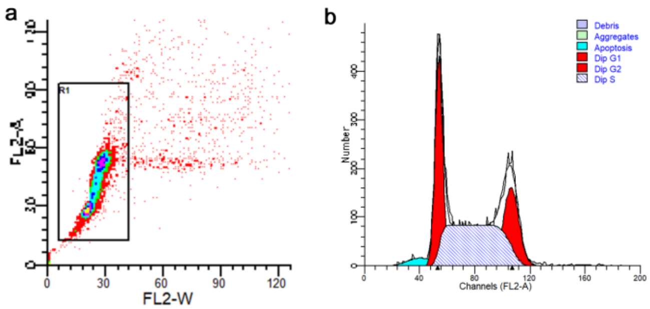

Gating strategy related to Fig. 7e.   Fig. 7e FH-CT

Supplementary Figure 5. A figure exemplifying the gating strategy used in Flow Cytometry related to Fig. 7e. a A figure showing the gating strategy used in Flow Cytometry related to Fig. 7e. **b** DNA contents distribution of the cells that gated in **a**.

Supplementary Figure 6. Uncropped scans of western blots for Fig. 1a & 1b.

Fig.1a

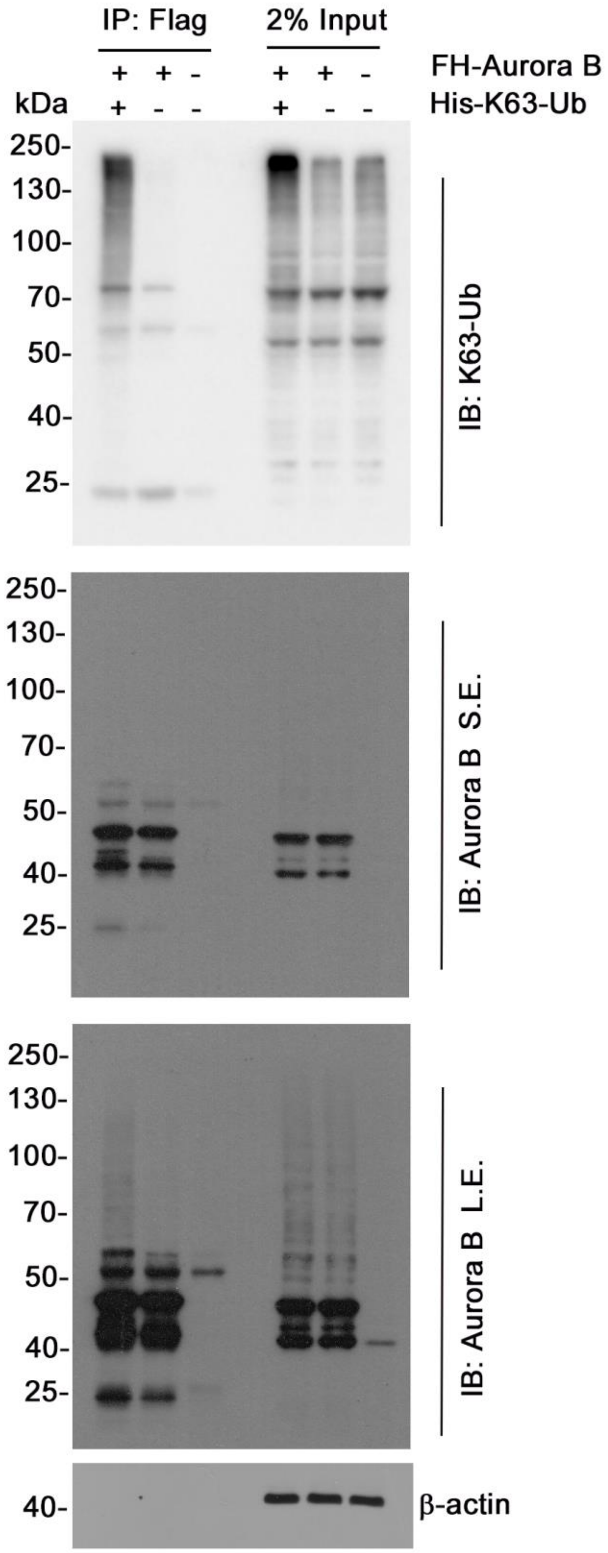

Fig.1b

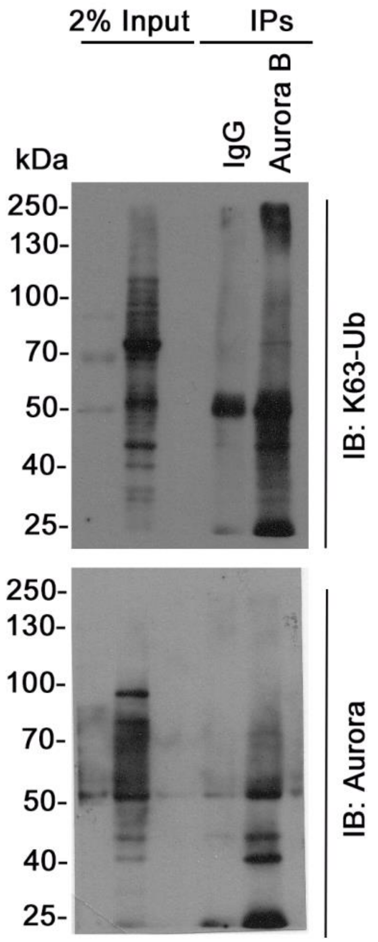

Supplementary Figure 7. Uncropped scans of western blots for Fig. 1c & 1d.

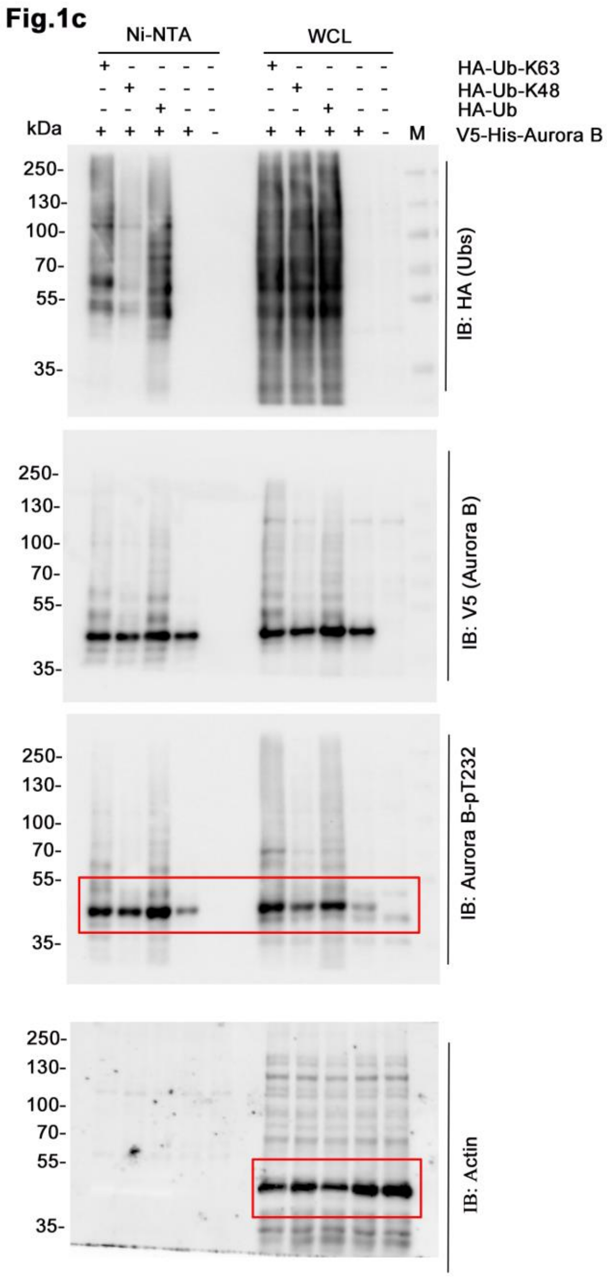

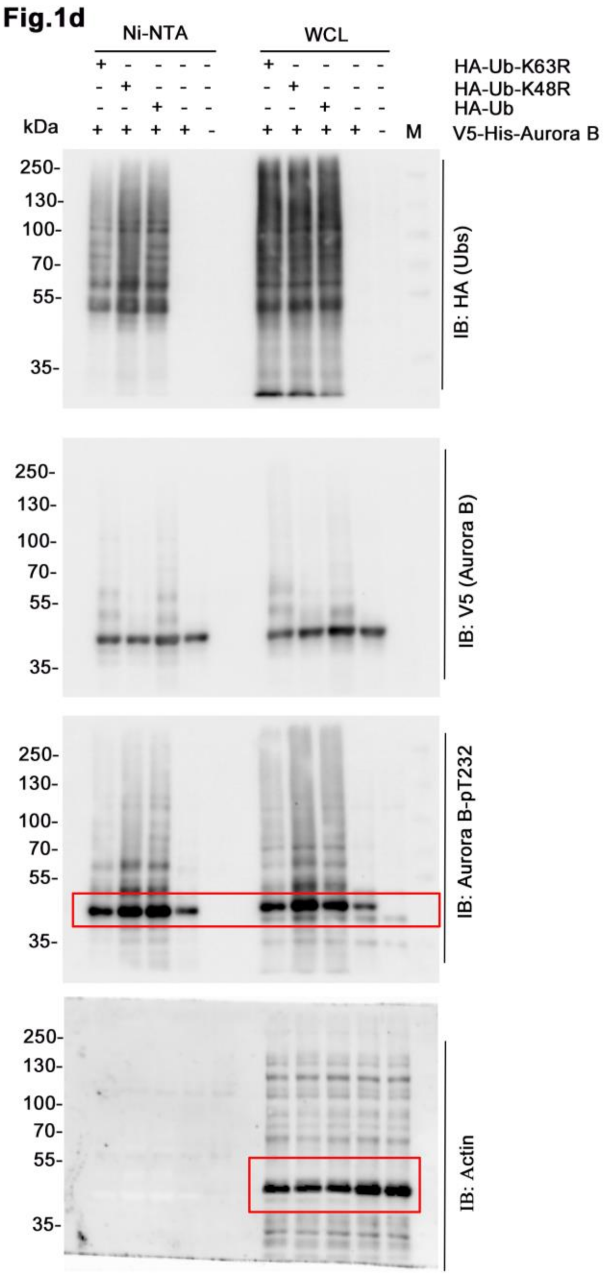

Supplementary Figure 8. Uncropped scans of western blots for Fig. 2a-2c.

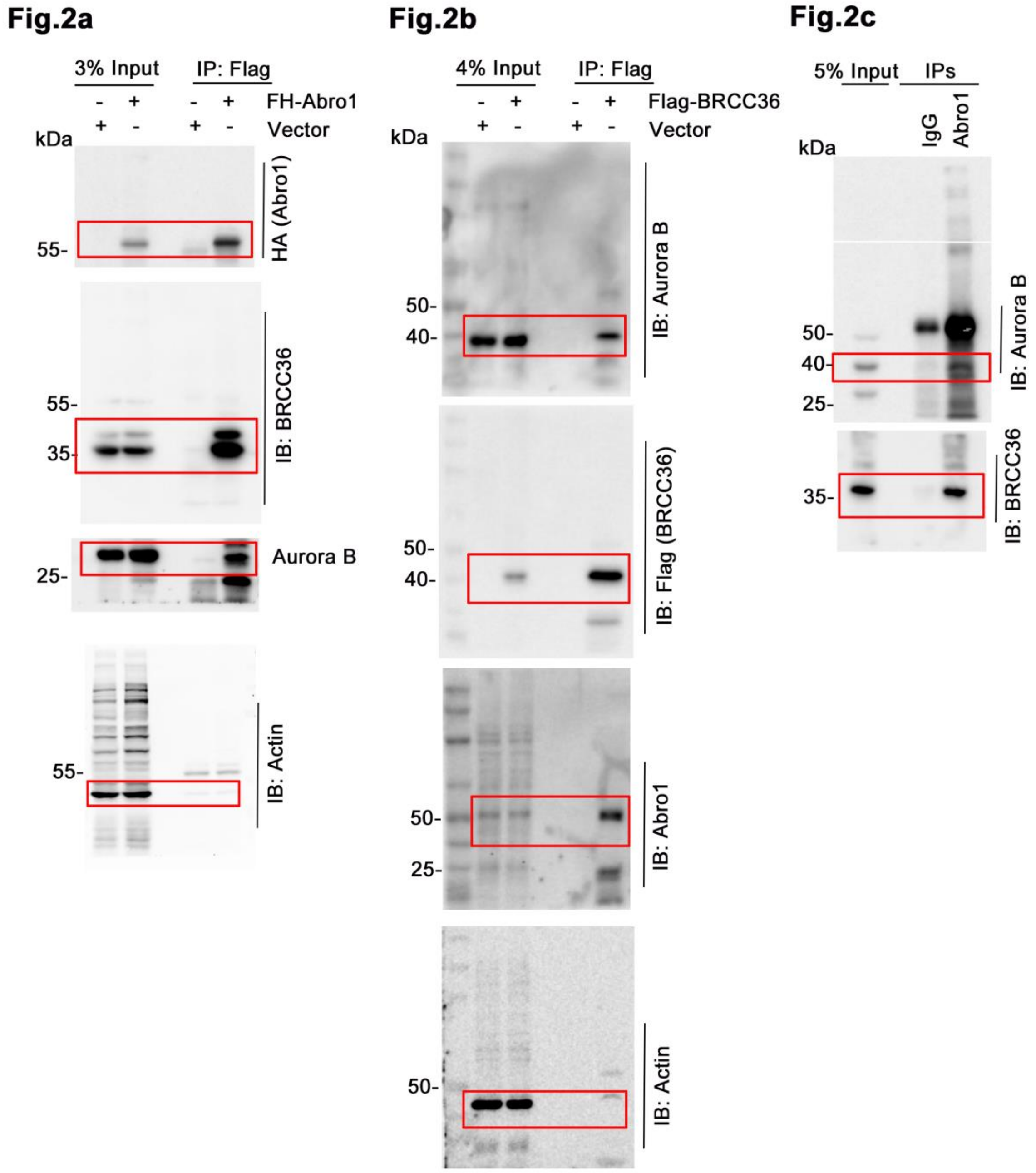

Supplementary Figure 9. Uncropped scans of western blots for Fig. 2d & 2e.

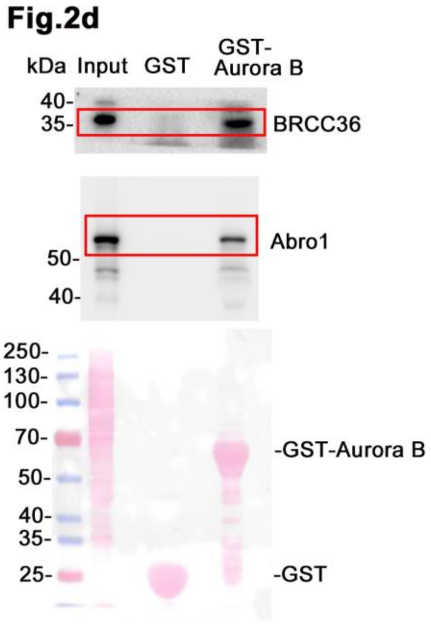

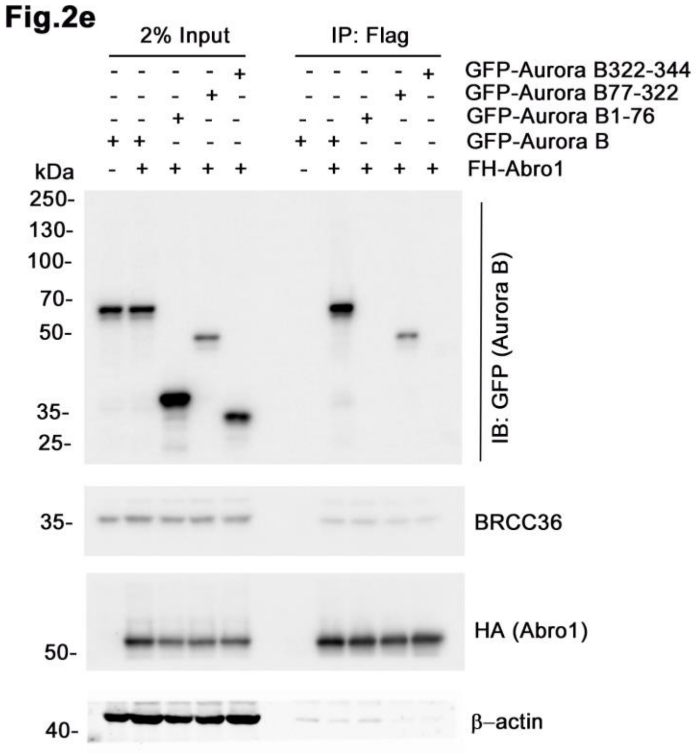

Supplementary Figure 10. Uncropped scans of western blots for Fig. 2h.

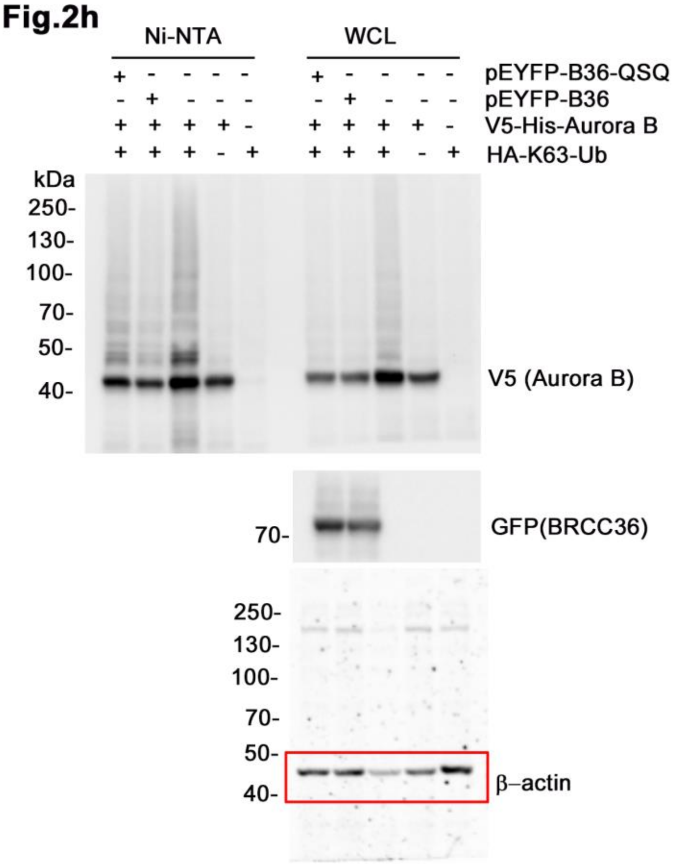

Supplementary Figure 11. Uncropped scans of western blots for Fig. 2i.

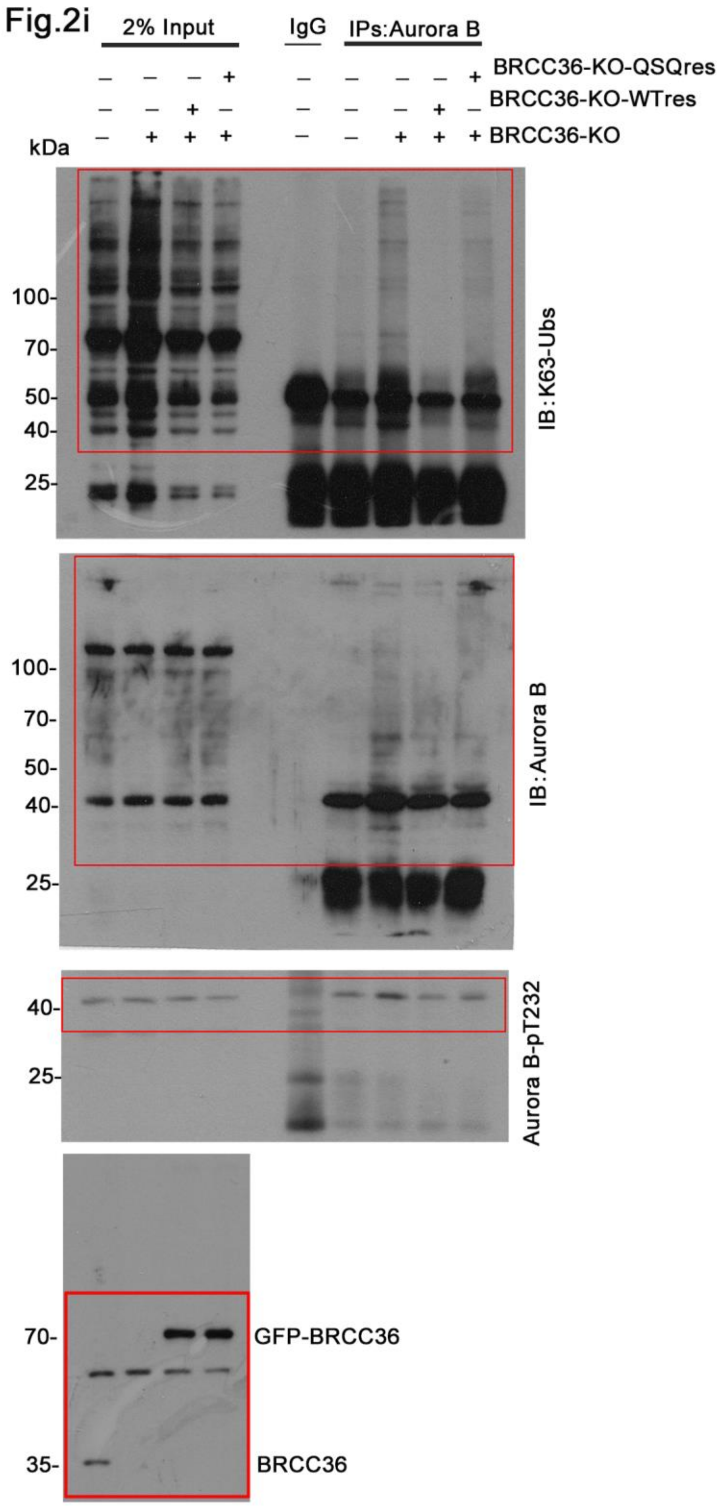

Supplementary Figure 12. Uncropped scans of western blots for Fig. 4a.

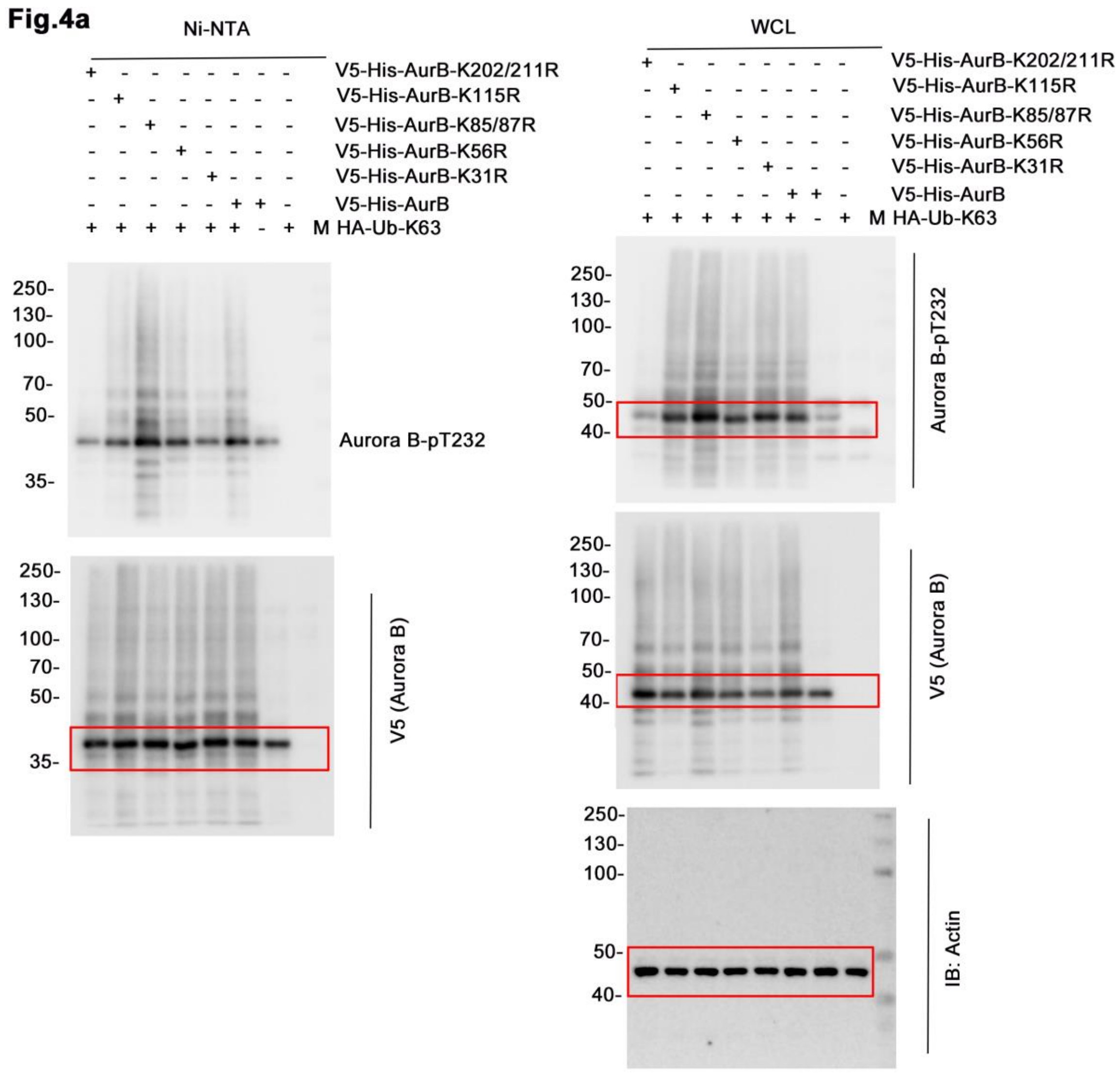

Supplementary Figure 13. Uncropped scans of western blots for Fig. 4b.

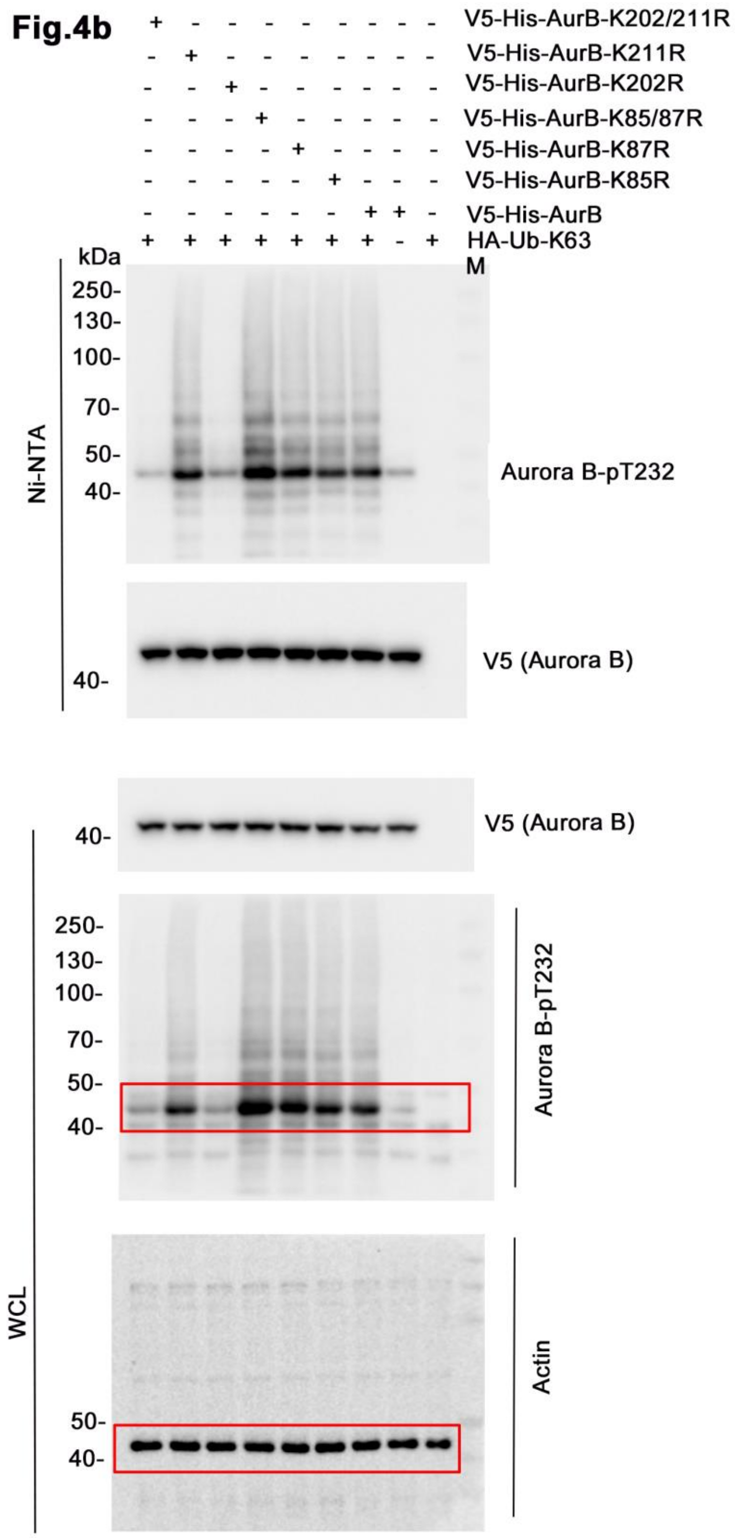

Supplementary Figure 14. Uncropped scans of western blots for Fig. 4d & 4i.

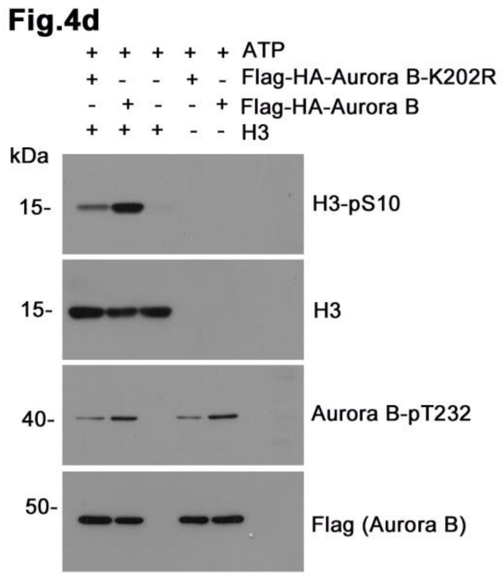

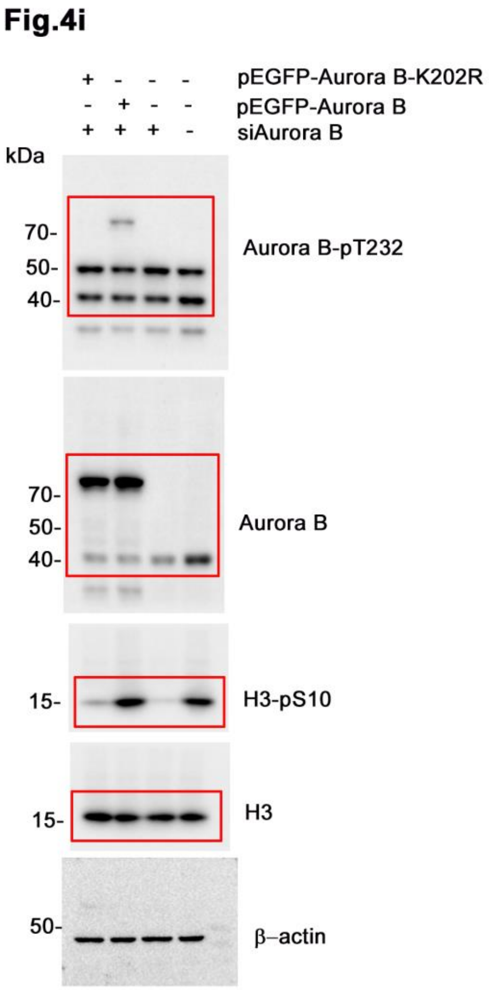

Supplementary Figure 15. Uncropped scans of western blots for Fig. 5a.

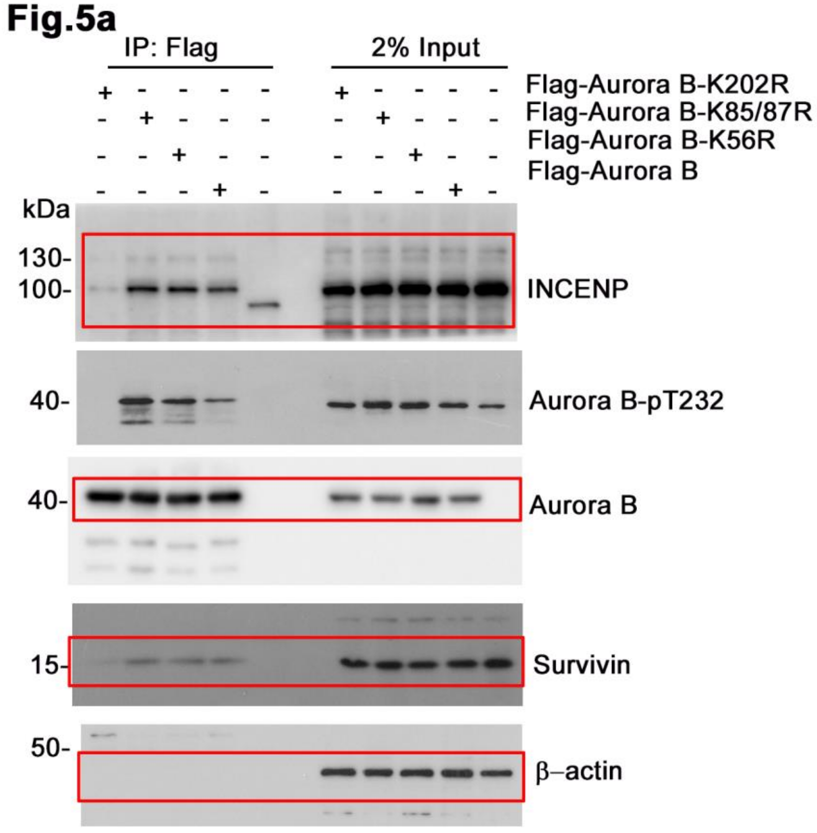

Supplementary Figure 16. Uncropped scans of western blots for Fig. 7a.

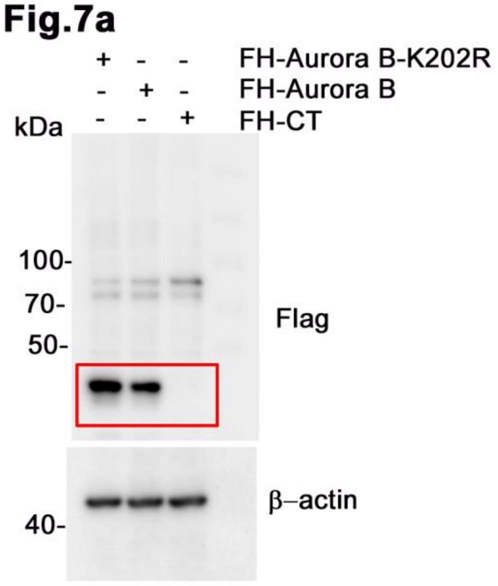

Supplementary Figure 17. Uncropped scans of western blots for FigS. 1b & 1c.

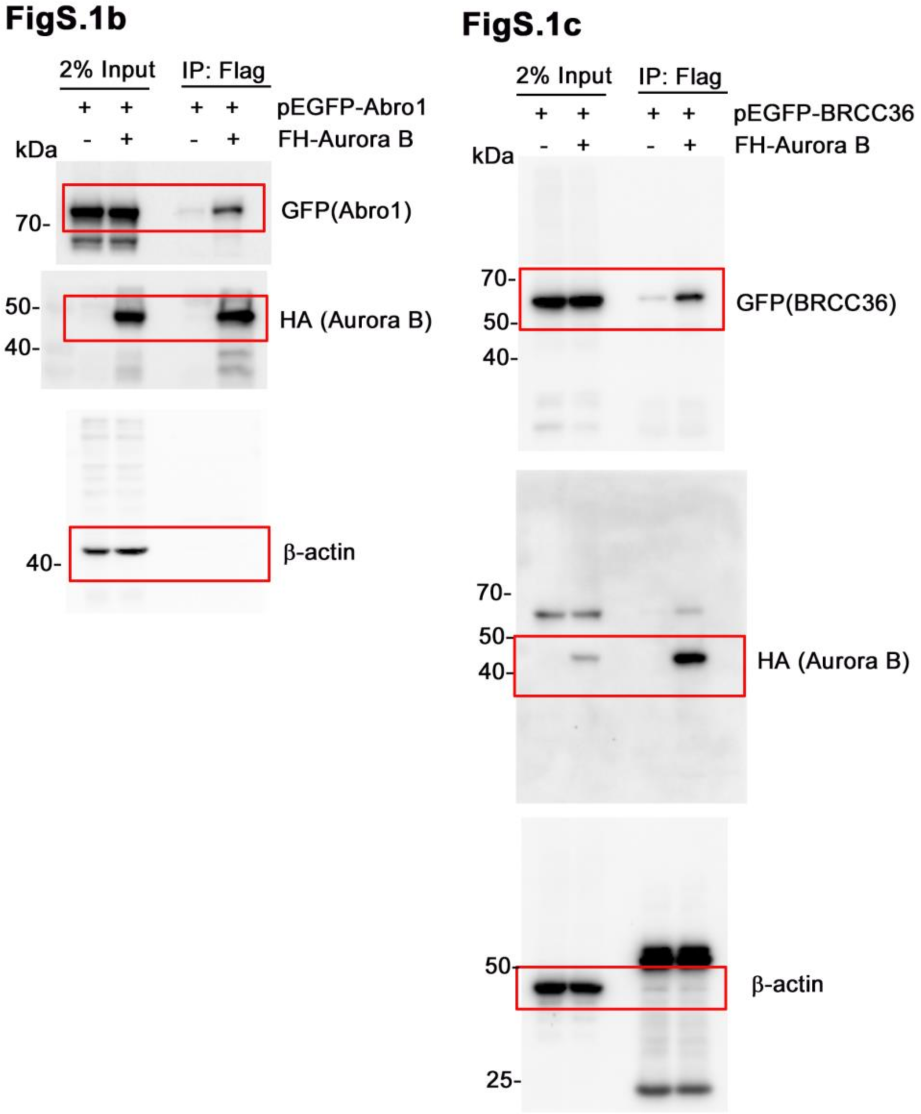

Supplementary Table S1. List of primary antibodies used for western blotting in this study.

Antibodies	Source	Dilution
**Primary antibodies used for Western Blotting**		
Rabbit anti-Aurora B #3094S	Cell Signaling Technology	1:1000
Mouse anti-β-actin # sc-47778	Santa Cruz	1: 5000
Rabbit anti-Flag #4793S	Cell Signaling Technology	1:2000
Rabbit anti-GFP # ab290	Abcam	1:3000
Rabbit anti-H3 #BD-PM3414	Biodragon	1:5000
Rabbit anti-H3-pS10 #0701S	Cell Signaling Technology	1:1000
Rabbit anti-HA #3924S	Cell Signaling Technology	1:3000
Mouse anti-Hec1 #ab3613	Abcam	1:300
Rabbit anti-Hec1-pS55 #GTX70017	GeneTex	1:500
Rabbit anti-INCENP # ab12183	Abcam	1:1000
Rabbit anti-K63 specific Ub #5621S	Cell Signaling Technology	1:1000
Rabbit anti-Phospho-Aurora B (Thr232) #2914S	Cell Signaling Technology	1:1000
Rabbit anti-Survivin #2808T	Cell Signaling Technology	1:1000
Rabbit anti-V5 # ab182088	Abcam	1:1000
Rabbit anti-BRCC36;	Our lab (PMID: 26195665)	1:3000
Rabbit anti-Abro1	Our lab (PMID: 26195665)	1:2000
**Primary antibodies used for immunofluorescent (IF) staining**		
Human anti-centromere positive serum #15-235-0001	Antibodies Inc.	1:300
Mouse anti-Aurora B # 611082	BD Transduction Laboratories	1:200
Mouse anti-α-tubulin #3873S	Cell Signaling Technology	1:1000
Rabbit anti-Astrin #14726-1-AP	Proteintech	1:100
Rabbit anti-Aurora B pT232 #600-401-677T	Rockland.	1:100
Rabbit anti-H3-pS10 #0701S	Cell Signaling Technology	1:100
Rabbit anti-Hec1-pS55 #GTX70017	GeneTex	1:100

Rabbit anti-INCENP # ab12183	Abcam	1:1000
Rabbit anti-Mad1 #8322-1-AP	Proteintech	1:200
Rabbit anti-MCAK # ab187652	Abcam	1:200
Rabbit anti-Survivin #2808T	Cell Signaling Technology	1:1000

Supplementary Table S2. List of secondary antibodies used for immunofluorescent staining in this study.

Antibodies	Conjugation	Source	Dilution
Goat anti-Human IgG # A11013	Alexa Fluor 488	Invitrogen	1:200
Goat anti-human IgG # A21445	Alexa Fluor 647	Invitrogen	1:200
Goat anti-mouse IgG # A21058	Alexa Fluor 680	Invitrogen	1:200
Goat anti-mouse IgG # A32723	Alexa Fluor 488	Invitrogen	1:200
Goat anti-rabbit IgG # A21441	Alexa Fluor 488	Invitrogen	1:200
