# Supplementary material for: The deubiquitinating enzyme complex BRISC regulates Aurora B activation via lysine-63-linked ubiquitination in mitosis
Source: Commun Biol. 2022 Dec 6;5:1335. doi: 10.1038/s42003-022-04299-4 (PMC9726926; doi:10.1038/s42003-022-04299-4)
Supplement: Supplementary file 2 — Description of Additional Supplementary Files [file 42003_2022_4299_MOESM2_ESM.pdf]

Description of Additional Supplementary Files

File name: Supplementary Data 1

Description: The list of identified peptides/protein hits in the mass spectrometry, which is related to Figure 2.

File name: Supplementary Video 1

Description: Time-lapse imaging of dividing control siRNA–treated cells, related to Fig.6a.

File name: Supplementary Video 2

Description: Time-lapse imaging of dividing siAurora B-treated cells, related to Fig.6a.

File name: Supplementary Video 3

Description: Time-lapse imaging of dividing cells treated with siAurora B and rescued with pEGFP-Aurora B-WT, related to Fig. 6a.

File name: Supplementary Video 4

Description: Time-lapse imaging of dividing cells treated with siAurora B and rescued with pEGFP-Aurora B-K202R, related to Fig. 6a.
