# Supplementary material for: The deubiquitinating enzyme complex BRISC regulates Aurora B activation via lysine-63-linked ubiquitination in mitosis
Source: Commun Biol. 2022 Dec 6;5:1335. doi: 10.1038/s42003-022-04299-4 (PMC9726926; doi:10.1038/s42003-022-04299-4)
Supplement: Supplementary file 8 — Reporting Summary [file 42003_2022_4299_MOESM8_ESM.pdf]

nature portfolio

Corresponding author(s): Li Li

Last updated by author(s): Nov 23, 2022

Reporting Summary

Nature Portfolio wishes to improve the reproducibility of the work that we publish. This form provides structure for consistency and transparency in reporting. For further information on Nature Portfolio policies, see our Editorial Policies and the Editorial Policy Checklist.

Statistics

For all statistical analyses, confirm that the following items are present in the figure legend, table legend, main text, or Methods section.

n/a	Confirmed	
☐	☒	The exact sample size (*n*) for each experimental group/condition, given as a discrete number and unit of measurement
☐	☒	A statement on whether measurements were taken from distinct samples or whether the same sample was measured repeatedly
☐	☒	The statistical test(s) used AND whether they are one- or two-sided<br>*Only common tests should be described solely by name; describe more complex techniques in the Methods section.*
☒	☐	A description of all covariates tested
☒	☐	A description of any assumptions or corrections, such as tests of normality and adjustment for multiple comparisons
☐	☒	A full description of the statistical parameters including central tendency (e.g. means) or other basic estimates (e.g. regression coefficient) AND variation (e.g. standard deviation) or associated estimates of uncertainty (e.g. confidence intervals)
☐	☒	For null hypothesis testing, the test statistic (e.g. F, t, r) with confidence intervals, effect sizes, degrees of freedom and P value noted<br>*Give P values as exact values whenever suitable.*
☒	☐	For Bayesian analysis, information on the choice of priors and Markov chain Monte Carlo settings
☒	☐	For hierarchical and complex designs, identification of the appropriate level for tests and full reporting of outcomes
☐	☒	Estimates of effect sizes (e.g. Cohen's *d*, Pearson's *r*), indicating how they were calculated

Our web collection on statistics for biologists contains articles on many of the points above.

Software and code

Policy information about availability of computer code

Data collection	Primers were designed using oligo7; sgRNAs were designed using tools provided by chopchop.com web; Western blotting images were collected with the Chemidoc mp imaging system; 96-well plates were read using the Tecan software; Confocal images and live-cell videos were collected by LSM880, Zeiss or TCS-SP8 DIVE, Lecia; Colony formation images were caputured by GelDoc-It immaging system.
Data analysis	Confocal images collected by Zeiss LSM880 were analyzed by ZEN3.1, and processed by using Adobe Photoshop CC2018; Colony formation images were calculated by Image .J; Flow cytometry data was analyzed using the FACSDiva software; Statistical analyses were performed using the GraphPad Prism v8.0 Software.

For manuscripts utilizing custom algorithms or software that are central to the research but not yet described in published literature, software must be made available to editors and reviewers. We strongly encourage code deposition in a community repository (e.g. GitHub). See the Nature Portfolio guidelines for submitting code & software for further information.

Data

Policy information about availability of data

All manuscripts must include a data availability statement. This statement should provide the following information, where applicable:

- Accession codes, unique identifiers, or web links for publicly available datasets
- A description of any restrictions on data availability
- For clinical datasets or third party data, please ensure that the statement adheres to our policy

All data generated or analysed during this study are included in this published article and its supplementary information files. Uncropped scans of blots were provided as Supplementary Figures 6-17. The raw data based on LC-MS/MS were deposited at Figshare with a doi: 10.6084/m9.figshare.21541524. The Supplementary videos 1-4 related to Fig. 6a were deposited at Figshare with a doi: 10.6084/m9.figshare.21561786.

Human research participants

Policy information about studies involving human research participants and Sex and Gender in Research.

Reporting on sex and gender	*Use the terms sex (biological attribute) and gender (shaped by social and cultural circumstances) carefully in order to avoid confusing both terms. Indicate if findings apply to only one sex or gender; describe whether sex and gender were considered in study design whether sex and/or gender was determined based on self-reporting or assigned and methods used. Provide in the source data disaggregated sex and gender data where this information has been collected, and consent has been obtained for sharing of individual-level data; provide overall numbers in this Reporting Summary. Please state if this information has not been collected. Report sex- and gender-based analyses where performed, justify reasons for lack of sex- and gender-based analysis.*
Population characteristics	*Describe the covariate-relevant population characteristics of the human research participants (e.g. age, genotypic information, past and current diagnosis and treatment categories). If you filled out the behavioural & social sciences study design questions and have nothing to add here, write "See above."*
Recruitment	*Describe how participants were recruited. Outline any potential self-selection bias or other biases that may be present and how these are likely to impact results.*
Ethics oversight	*Identify the organization(s) that approved the study protocol.*

Note that full information on the approval of the study protocol must also be provided in the manuscript.

Field-specific reporting

Please select the one below that is the best fit for your research. If you are not sure, read the appropriate sections before making your selection.

☒ Life sciences ☐ Behavioural & social sciences ☐ Ecological, evolutionary & environmental sciences

For a reference copy of the document with all sections, see nature.com/documents/nr-reporting-summary-flat.pdf

Life sciences study design

All studies must disclose on these points even when the disclosure is negative.

Sample size	For Fig. 3a-3f, Fig. 4e-4h & Fig. 5b-5e, signals were collected from 15 cells, and 6 areas for each cell were calculated; Fig. 3g, 3h & 3i, n=30 cells and five paired kinetochores for each cell were calculated ; Fig. 3j, n=20cells and five paired kinetochores for each cell were calculated; Fig. 3k & 3l, n=15 cells. For Fig. 6a-6c, n=8-12cells. For Fig. 6d & 6e, data from 3 independent experiments, 30 cells were calculated each time; for Fig. 6f & 6g, data from 3 independent experiments, 20 cells were calculated each time; for Fig. 6h & 6i, data from 3 independent experiments, 15 cells were calculated each time. For Fig. 7b-7g, data from 3 independent experiments, and 3 technical replicates each time for Fig. 7b & 7c and Fig. 7e & 7f, 6 technical replicates each time for Fig. 7d & 7g.
Data exclusions	No data was excluded.
Replication	All the experiments listed above were repeated at least three times.
Randomization	For relative immunofluorescence intensity analysis in Fig.3a-3e, Fig. 4e-4h & Fig. 5b-5e, the area used for calculation were selected randomly.
Blinding	The investigators were blinded during data collection for the flow cytometry experiments and confocal image capture. Blinding was not relevant in the rest of the experiments, since measures are taken automatically with no human intervention.

Reporting for specific materials, systems and methods

We require information from authors about some types of materials, experimental systems and methods used in many studies. Here, indicate whether each material, system or method listed is relevant to your study. If you are not sure if a list item applies to your research, read the appropriate section before selecting a response.

Materials & experimental systems

n/a	Involved in the study
☐	☒ Antibodies
☐	☒ Eukaryotic cell lines
☒	☐ Palaeontology and archaeology
☒	☐ Animals and other organisms
☒	☐ Clinical data
☒	☐ Dual use research of concern

Methods

n/a	Involved in the study
☒	☐ ChIP-seq
☐	☒ Flow cytometry
☒	☐ MRI-based neuroimaging

Antibodies

Antibodies used	Antibodies used in this study were listed in Supplementary Table S1 & S2.
Validation	The rabbit anti-BRCC36 antibody and anti-Abro1 antibody have been validated in the paper PMID: 26195665. All the other antibodies used have been validated by the vendors.

Eukaryotic cell lines

Policy information about cell lines and Sex and Gender in Research

Cell line source(s)	The HEK293T cell line and HeLa cell line used in this study were purchased from ATCC.
Authentication	The HEK293T cell line used was authenticated by Shanghai BiowingApplied Biotechnology Co. Ltd.
Mycoplasma contamination	All the cell lines used in this study were routinely tested for mycoplasma contamination and negative for mycoplasma contamination.
Commonly misidentified lines (See ICLAC register)	No commonly misidentified lines were used.

Flow Cytometry

Plots

Confirm that:

☒ The axis labels state the marker and fluorochrome used (e.g. CD4-FITC).

☒ The axis scales are clearly visible. Include numbers along axes only for bottom left plot of group (a 'group' is an analysis of identical markers).

☒ All plots are contour plots with outliers or pseudocolor plots.

☒ A numerical value for number of cells or percentage (with statistics) is provided.

Methodology

Sample preparation	Human cervical carcinoma HeLa cells were infected with the Aurora B or Aurora B-mutant virus for 96h. Then, cells were fixed with 75% cold ethanol for overnight, digested with RNase A for 30min, and rinsed with PBS. Suspend the cell pellets in 1mL of PI labeling solution and submit to flow cytometry.
Instrument	BD FACSCanto™ II system.
Software	ModFitLT V3.0
Cell population abundance	Flow cytometry was used for quantification purposes only, and no post-sorting fractions were collected.

Gating strategy

First, a pulse width-pulse area plot was used for gating to select a single cell population. Then this gate was applied to the scatter plot and excluded obvious cellular debris. Combine the two gates and apply them to the PI histogram. A figure exemplifying the gating strategy was provided as Supplementary Figure 5.

☒ Tick this box to confirm that a figure exemplifying the gating strategy is provided in the Supplementary Information.
